# Frizzled BRET sensors based on bioorthogonal labeling of unnatural amino acids reveal WNT-induced dynamics of the cysteine-rich domain

**DOI:** 10.1126/sciadv.abj7917

**Published:** 2021-11-10

**Authors:** Maria Kowalski-Jahn, Hannes Schihada, Ainoleena Turku, Thomas Huber, Thomas P. Sakmar, Gunnar Schulte

**Affiliations:** 1 Karolinska Institutet, Department of Physiology and Pharmacology, Section of Receptor Biology and Signaling, Biomedicum 6D, S-17165 Stockholm, Sweden.; 2 Laboratory of Chemical Biology and Signal Transduction, The Rockefeller University, 1230 York Ave., New York, NY 10065, USA.; 3 Karolinska Institutet, Department of Neurobiology, Care Sciences and Society, Center for Alzheimer Research, Division of Neurogeriatrics, S-17164 Stockholm, Sweden.

## Abstract

Frizzleds (FZD_1–10_) are G protein–coupled receptors containing an extracellular cysteine-rich domain (CRD) binding Wingless/Int-1 lipoglycoproteins (WNTs). Despite the role of WNT/FZD signaling in health and disease, our understanding of how WNT binding is translated into receptor activation and transmembrane signaling remains limited. Current hypotheses dispute the roles for conformational dynamics. To clarify how WNT binding to FZD translates into receptor dynamics, we devised conformational FZD-CRD biosensors based on bioluminescence resonance energy transfer (BRET). Using FZD with N-terminal nanoluciferase (Nluc) and fluorescently labeled unnatural amino acids in the linker domain and extracellular loop 3, we show that WNT-3A and WNT-5A induce similar CRD conformational rearrangements despite promoting distinct signaling pathways and that CRD dynamics are not required for WNT/β-catenin signaling. Thus, these FZD-CRD biosensors provide insights into binding, activation, and signaling processes in FZDs. The sensor design is broadly applicable to explore ligand-induced dynamics also in other membrane receptors.

## Teaser

WNTs induce conformational changes in the extracellular CRD of Frizzleds irrespective of their signaling profile.

## INTRODUCTION

The class Frizzled (FZD) of G protein–coupled receptors (GPCRs), also known as class F, comprises 10 FZD subtypes (FZD_1–10_) and Smoothened (SMO) (*1*). These cell surface membrane proteins share the structural hallmarks of GPCRs [extracellular N terminus, seven membrane-spanning helices, transmembrane domain 1 (TM1) to TM7, connected via three extracellular and three intracellular loops, ECL1 to ECL3 and ICL1 to ICL3, and an intracellular C terminus]. FZDs also display a class-typical cysteine-rich domain (CRD) at an extended N terminus, which is crucial for the engagement of their endogenous ligands with the receptor (*2*).

While SMO regulates Hedgehog signaling, FZDs bind extracellular, secreted lipoglycoproteins of the Wingless/Int-1 family (WNTs) and mediate the vital effects of these 19 mammalian WNT paralogues on cellular proliferation, migration, differentiation, tissue polarity, tissue homeostasis, and cancer development (*3*). On the one hand, FZDs mediate signaling through dishevelled (DVL1 to DVL3)–dependent pathways resulting in either the stabilization and nuclear translocation of β-catenin or planar cell polarity-like signaling (PCP) (*4*, *5*). On the other hand, they mediate DVL-independent signaling through heterotrimeric G proteins (*6*–*8*). Despite the enormous physiological relevance of the WNT/FZD signaling system in human health and disease, little is known about ligand/receptor selectivity, the molecular details that underlie receptor activation or the initiation of intracellular signaling. Thus, it remains unclear how distinct WNT/FZD complexes achieve pathway selectivity (*9*–*11*).

Given the lack of structural information on WNT/FZD complexes and the fact that WNTs can bind to purified FZD-CRDs without the presence of the transmembrane core of the receptor (*2*), uncertainty remains about the structural basis for agonist-induced, FZD-dependent signaling. As of today, two main models emerge, explaining how FZDs translate WNT/CRD association into different cellular signaling branches. One hypothesis is based on WNT-mediated cross-linking of FZDs with co-receptors [e.g., with low-density lipoprotein receptor-related protein 5 or 6 (LRP5/6), reversion-inducing cysteine-rich protein with Kazal motifs (RECK), tyrosine-protein kinase transmembrane receptor ROR2, or the adhesion GPCR ADGRA2, also known as GPR124; (*12*)], determining which intracellular transducer protein is recruited to subsequently convey the stimulus to the cell interior. This “signalosome” concept is widely accepted for signaling of FZDs through β-catenin, which depends on WNT-induced interaction of FZDs with LRP5/6 (*2*, *13*, *14*), and would allow for conformational flexibility of the extracellular linker region between the CRD and the receptor’s transmembrane core (*15*). On the other hand, WNT-induced conformational changes in FZDs could also independently from WNT co-receptors promote distinct cellular signaling pathways (*8*, *16*–*20*). The latter model mirrors the concept of how class A and B GPCRs activate, for instance, heterotrimeric G proteins in response to a ligand-induced opening of the intracellular receptor surface and subsequent GPCR/G protein coupling (*21*). However, this process would require a constrained conformational mobility of the linker region to allow for mutual allosteric regulation of WNT binding and transducer coupling according to the ternary complex model (*21*, *22*).

Although WNT-3A/β-catenin signaling occurs independently from heterotrimeric G proteins in human embryonic kidney (HEK) 293 cells (*6*), it remains obscure whether and how the signalosome and “ternary complex” model are mechanistically intertwined in FZDs, hampering a rational development of FZD subtype–specific and intracellular pathway–selective drugs to treat WNT/FZD-dependent disorders such as colon and pancreatic cancer.

Recent studies provided intriguing but controversial insights into different aspects of the WNT/FZD system including the question of ligand/receptor selectivity and the underlying activation mechanism of FZDs (*9*, *15*, *20*, *23*). For instance, mutagenesis-based approaches suggested that FZD_5_ does not undergo conformational changes while signaling through DVL/β-catenin (*15*), molecular dynamics (MD) simulations of FZD_4_ (*19*), and experiments with conformational FZD biosensors revealed structural flexibility and its importance for downstream signaling mediated by these receptors (*8*, *16*, *18*, *20*). These conflicting findings highlight the need for advanced biophysical approaches and molecular tools to investigate WNT/FZD interaction and the conformational landscape of FZDs to better understand the FZD mode of action.

The assessment of conformational dynamics of GPCRs in living cells was facilitated by the design of fluorescence resonance energy transfer (FRET)– and bioluminescence resonance energy transfer (BRET)–based biosensors already in the early 2000s (*24*, *25*). Their development has not only allowed to monitor the structural rearrangements of various GPCRs—including FZD_5_ and FZD_6_ (*16*, *18*)—in real time and single cells, but refinements of the sensor design have further enabled the establishment of screening-compatible assay formats (*26*, *27*). More recently, a distinct approach based on conformationally sensitive, circularly permutated fluorescent proteins, which were introduced to various GPCRs to monitor receptor activation in living animals (*28*), provided unprecedented insights into WNT-induced conformational dynamics of FZDs (*20*). While these conformational GPCR sensors exclusively detect the receptors’ structural dynamics at the intracellular side, several FRET-based GPCR sensors have been devised to study the extracellular conformational rearrangements of mainly class C GPCRs labeled using SNAP- and CLIP-tag technology (*29*–*31*). In addition, genetic code expansion and labeling of unnatural amino acids (uaas) enabled the investigation of tethered agonist (“Stachel”) exposure in class adhesion GPCRs (*32*).

In class F GPCRs, MD simulations based on an inactive SMO crystal structure (including a resolved CRD) revealed moderate CRD flexibility in the absence of a ligand, which is further restrained upon cholesterol binding to the CRD (*33*). Similarly, MD studies on an active SMO structure show only minor CRD movement when bound to cholesterol on the CRD and the receptor core, allowing the receptor to adapt an active TM7 conformation to promote intracellular signaling (*34*). However, the CRD-binding ligands of SMO and FZDs are distinctively different (cholesterol versus WNTs, respectively), and the linker domain between the CRD and the 7TM core of the receptor is notably shorter in SMO than in FZDs (*1*), indicating that the functional dynamics of the CRD might also differ. FZD structures including a fully resolved CRD are not available, and techniques to investigate extracellular dynamics of GPCRs in intact cells are limited by the size of fluorescent tags. Inserting, for instance, a fluorescent protein in one of the receptor’s ECLs would most likely impair a proper folding of the receptor and thereby abolish its expression at the cell surface or could sterically interfere with ligand/receptor association.

To overcome these limitations of conventional fluorescent-labeling techniques, we linked small fluorescent probes to modified receptor residues using a minimally invasive labeling procedure based on genetic code expansion and incorporation of uaas serving as anchors for a subsequent bioorthogonal coupling reaction [strain-promoted inverse electron-demand Diels-Alder cycloaddition (SPIEDAC)] (*35*–*38*). With the aim to monitor and understand the WNT-induced dynamics of the extracellular CRD and its role for signal initiation, and to avoid interference with WNT-binding upon incorporation of bulky tags, we developed a set of conformational biosensors based on BRET between the small nanoluciferase (Nluc) and a fluorescently labeled uaa. The minimal size of the energy acceptor maintained functionality and ligand binding to the receptor and allowed assessment of WNT-induced dynamics of the FZD extracellular domains in living cells, providing intriguing insights into the mechanistic and kinetic details of receptor activation preceding WNT/FZD signaling.

## RESULTS

### MD simulations of a FZD_6_ model reveal CRD mobility

**Fig. 1.**
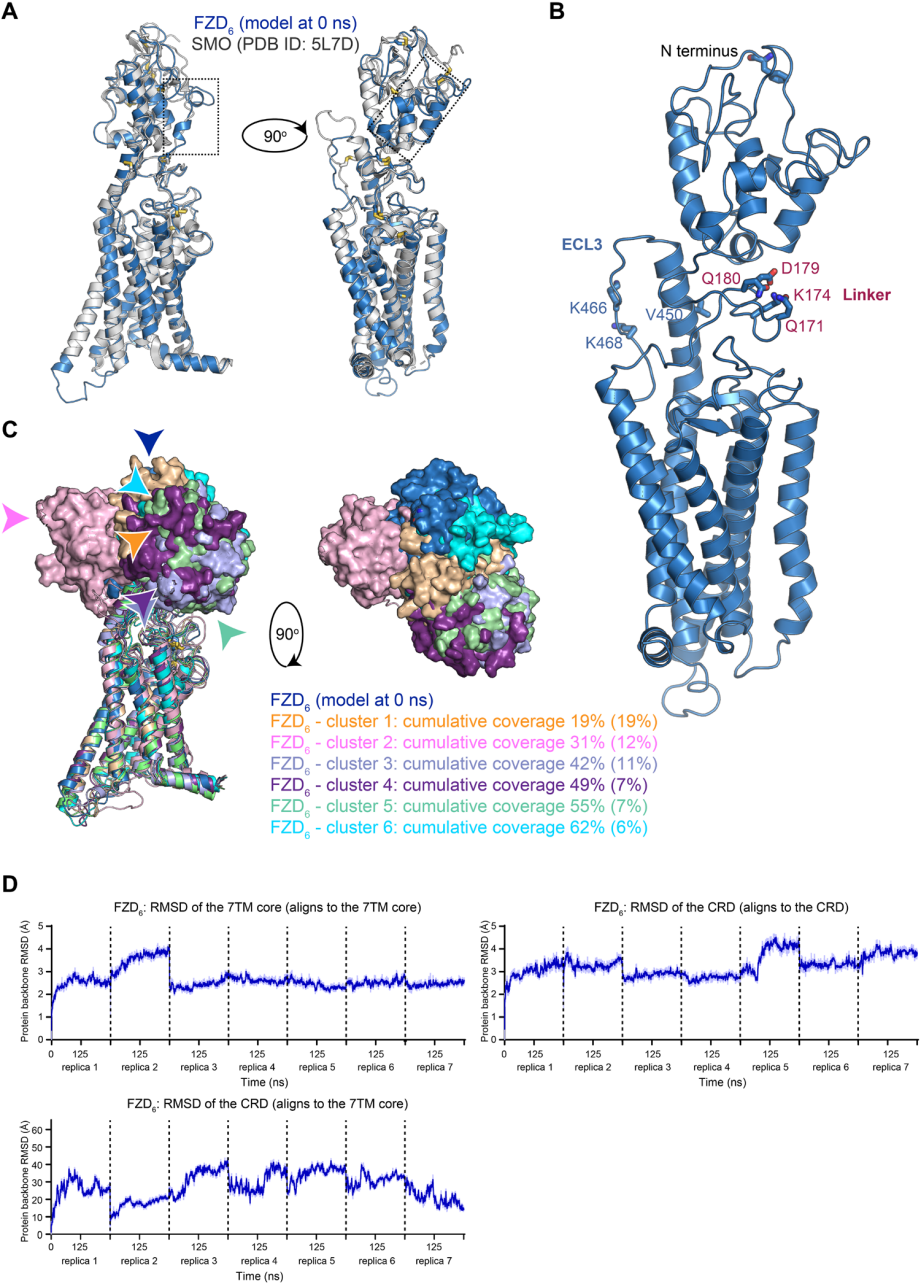
MD of FZD_6_ and receptor model with amber codon placement. (**A**) Superimposition of the FZD_6_ model (blue) and inactive SMO (gray; PDB ID: 5L7D). Disulfide bridges are shown as sticks. The dotted rectangles mark the extended linker region, which differs between these two structures. (**B**) A closer view to the FZD_6_ model. Amino acid residues selected for the point mutations are marked as sticks. Color code is as follows: red, oxygen; dark blue, nitrogen; light blue, carbon; yellow, sulfur. (**C**) The CRD movements in the MD simulation. The receptor cores are shown as cartoon and CRDs as solid surfaces. Colored arrows mark the locations of the N termini of each conformation cluster. Cumulative percentage of the time of coverage for each position cluster in the MD (absolute percentage for each cluster in parentheses). (**D**) Protein backbone root mean square deviations (RMSDs) of the receptor core (amino acids 160 to 511) and the CRD (amino acids 1 to 130) plotted as a continuous simulation trajectory. Dotted lines mark the independent simulation replicas. Thick blue traces indicate the moving average smoothed over a 2-ns window, and thin traces indicate the raw data.

To assess the putative range of motion of the CRD relative to the receptor core, we used first in silico approaches. In the absence of a self-evident template for the extended linker region of FZDs, we prepared an inactive full-length model of FZD_6_ using the iTasser server (*39*–*41*). FZD_6_ was selected for the modeling as it has a shorter extended linker sequence than other representatives of the four FZD homology clusters (i.e., FZD_4_, FZD_5_, and FZD_7_). The best-ranked model differs from the inactive SMO [Protein Data Bank (PDB) ID: 5L7D] only by this extended linker region (Fig. 1A) and was then used to initiate all-atom MD simulations (250 ns in seven independent replicas). In these simulations, the CRD probes a range of movements culminating to six main CRD orientations, which represent 62% of the simulation trajectory (Fig. 1C). Of these, clusters 2 and 5 mark the conformational extremes of the CRD movements of the whole trajectory. The 7TM core including the disulfide bond–stabilized part of the linker and the extended TM6 are stable throughout the simulation. Furthermore, the CRD remains stably folded, and only its location relative to the receptor core is changing (Fig. 1, C and D). Together, the MD data suggest that the CRD of FZD_6_ is capable to occupy distinct—conformationally restricted—orientations. To investigate whether these findings relate to WNT binding, we set out to study how the overall orientation of the CRD relative to the receptor core is affected by WNT stimulation using BRET technology.

### Incorporation of TCO*K into class F GPCRs and bioorthogonal labeling

As representatives of class F, we chose FZD_6_ and FZD_5_, which belong to different homology clusters of class F (*1*). The receptors were fused to an N-terminal Nluc epitope, following the 5-HT_3_ receptor signal peptide, and a C-terminal 1D4 epitope. By using the amber codon suppression technology, the uaa *trans*-cyclooct-2-ene-l-lysine (TCO*K) was introduced at distinct positions in the linker domain and the ECL3 of FZDs. We selected suitable positions for incorporation of TCO*K from the FZD_6_ simulation trajectory (Fig. 1B and fig. S1). The residues that were intended to be mutated were found to be in a distance toward the FZD’s N terminus that allows BRET analysis.

**Fig. 2.**
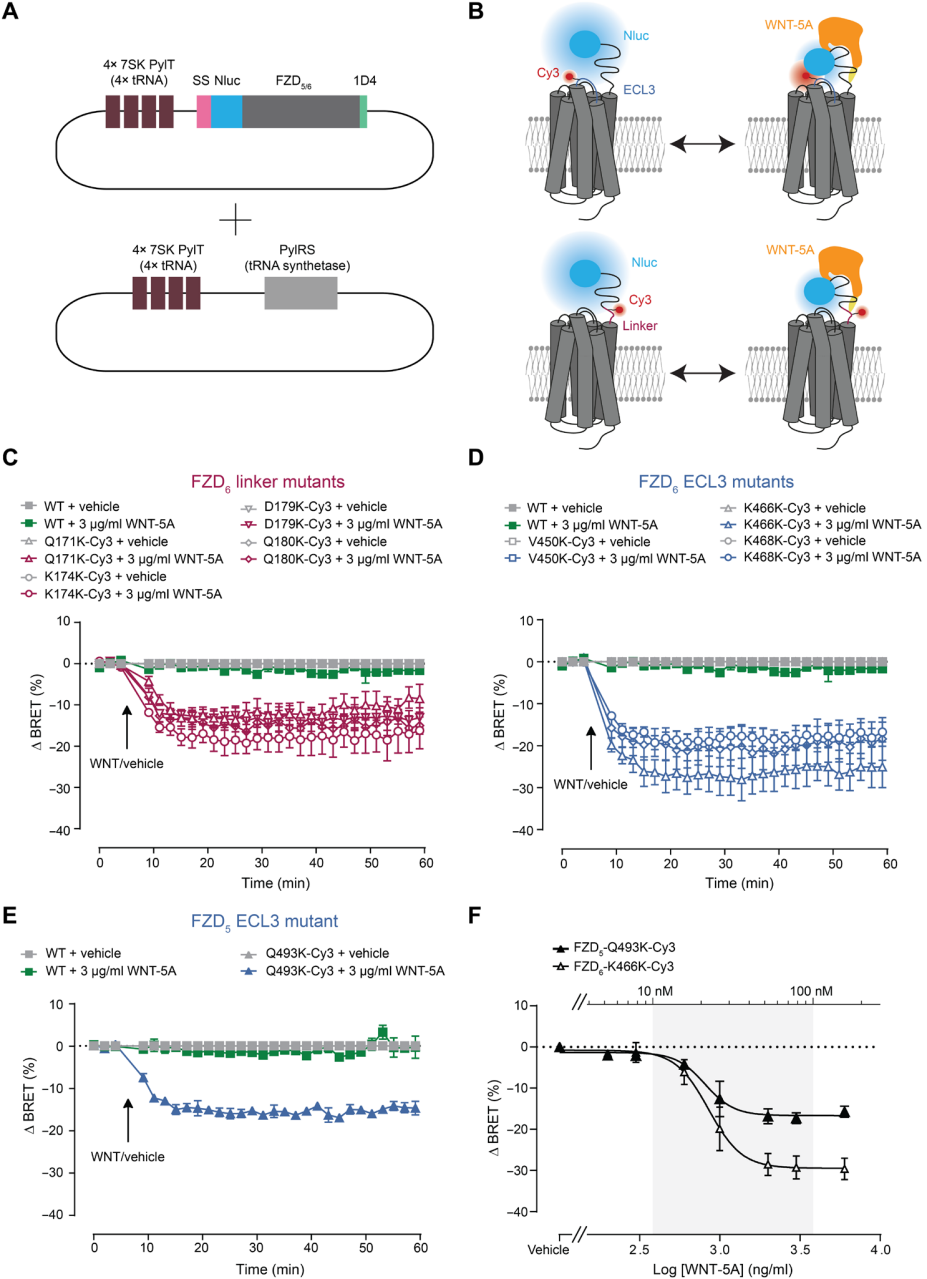
CRD rearrangements in FZD_5_ and FZD_6_ in response to WNT-5A. (**A**) Schematic depiction of cotransfected plasmids, one plasmid carrying four orthogonal tRNA repeats (4× 7SK PylT, Simon Elsässer, Addgene number: 140008) and amber codon–mutated FZD_5_ or FZD_6_ carrying a 5-HT_3_ receptor signal peptide, an N-terminal Nluc, and a C-terminal 1D4 epitope tag, and the other plasmid carrying four orthogonal tRNA repeats and the tRNA synthetase (*68*) (Addgene number: 140023). (**B**) Schematic depiction of the CRD sensor design with the N-terminally Nluc-tagged FZD. Fluorescence labeling of residues in the linker (pink) or ECL3 (blue) region of the receptor with Tet-Cy3. (**C** and **D**) BRET responses of FZD_6_ linker (C) and ECL3 (D) mutant CRD sensors upon WNT-5A treatment (3 μg/ml) or vehicle control. The arrow indicates the time point of WNT/vehicle application. (**E**) BRET responses of FZD_5_ ECL3 mutant CRD sensors upon WNT-5A treatment (3 μg/ml) or vehicle control. The arrow indicates the time point of WNT/vehicle application. (**F**) Concentration-response curves of WNT-5A on HEK293T cells expressing FZD_5_-Q493K-Cy3 or FZD6-K466K-Cy3. Concentration-response curves are normalized to vehicle control. All experiments were performed in HEK293T cells cotransfected with the indicated FZD_5_ or FZD_6_ sensors and the orthogonal tRNA/synthetase pair. SS, signal sequence; TCO*K, TCO-lysine.

For amber codon suppression, HEK293T cells were transfected with the amber-mutated receptor, and the corresponding orthogonal suppressor transfer RNA (tRNA)/aminoacyl tRNA synthetase pair in the presence of the uaa TCO*K (Fig. 2A). Western blot analysis using the monoclonal antibody (mAb) 1D4, which recognizes a fused C-terminal epitope tag, showed that the amber codon suppression with the uaa TCO*K was efficient (fig. S2A). Cell surface expression of all TCO*K-incorporated FZD mutants was determined using whole-cell enzyme-linked immunosorbent assay (ELISA) detecting the N-terminal Nluc epitope (fig. S2B). All mutants were expressed at the cell surface of HEK293T cells, averaging 31 to 81% compared with the respective wild-type (WT) receptor (fig. S2B).

In a next step, the receptor mutants were expressed in HEK293T cells, and living cells were labeled with the tetrazine (Tet)–bearing, membrane-impermeable fluorescent dye Tet-Cy3 (fig. S3A) using the SPIEDAC reaction. As an extension, also the membrane-permeable dye Tet–BODIPY-FL (BDP-FL) was used to label selected receptor mutants (fig. S4A). For quantification of the labeling efficiency of the different receptor mutants, a plate reader assay was used to detect fluorescence intensities (figs. S3B and S4B). While Tet-Cy3 specifically labeled HEK293T cells expressing receptor amber mutants exclusively in the presence of the uaa TCO*K, Tet–BDP-FL showed more unspecific labeling properties, likely owing to its high lipophilicity and membrane permeability, resulting in off-target labeling of TCO*K residues in intracellular compartments (including TCO*K-tRNA), other proteins with amber stop codons, and mature and immature FZDs (fig. S4B). In general, cell surface expression levels of the receptor mutants correlated with the labeling efficiency.

### WNT-5A induced BRET changes in the FZD_5_ and FZD_6_ CRD sensors

We took advantage of the N-terminally fused Nluc and the fluorescent dye, incorporated site-specifically in the linker region or ECL3 of the receptor, to establish BRET biosensors that can detect WNT-induced conformational rearrangements of the CRD (Fig. 2B). Initially, we tested all FZD_6_ CRD sensors, comprising four receptor mutants in the linker region and three receptor mutants in ECL3, in a ligand-free condition in terms of basal energy transfer between Nluc and the incorporated Cy3 or BDP-FL, respectively (fig. S5). Bioluminescence emission spectra of Nluc-tagged FZD_5_ and FZD_6_ CRD sensors, labeled with Tet–BDP-FL (green) or Tet-Cy3 (red), were recorded and normalized to the maximal Nluc emission. To exclude an unspecific fluorescent labeling of HEK293T cells expressing the receptor mutant sensors, we subtracted the bioluminescence signal obtained in cells expressing the WT receptor lacking incorporated TCO*K. We obtained emission peaks at ~510 nm for BDP-FL and ~570 nm for Cy3, respectively, with highest peaks for the FZD_6_-K466TCO*K mutant.

After confirming the occurrence of intramolecular basal BRET for all mutants, we applied WNT-5A (3 μg/ml), which corresponds to 70.86 nM (predicted molar mass of WNT-5A according to the supplier: 38,000 Da), to HEK293T cells expressing the different FZD CRD sensors and recorded the resulting BRET response of the Cy3-labeled FZD sensors over time. We detected dynamic WNT-induced BRET changes for all FZD_6_ linker (Fig. 2C) and ECL3 (Fig. 2D) mutant sensors with fitted maximal ∆BRET amplitudes varying between −11.61 ± 0.51% for the linker mutant FZD_6_-Q171K-Cy3 and −26.40 ± 0.74% for the ECL3 mutant FZD_6_-K466K-Cy3 (table S1).

We extended our studies to FZD_5_, which, in contrast to FZD_6_, mediates both G protein– and WNT/β-catenin–dependent signaling (*18*, *42*), and generated the ECL3 mutant FZD_5_-Q493TCO*K corresponding to FZD_6_-K466TCO*K. This FZD_5_ CRD sensor also displayed a WNT-induced conformational change of the CRD (Fig. 2E) with a maximal ∆BRET amplitude of −15.60 ± 0.23% [ΔBRET amplitudes in Fig. 2 (C to E) were fitted with a plateau after decay equation; see table S1].

A distant member of the class F GPCRs is the Hedgehog signal–mediating SMO, which is structurally related to FZD but, in contrast, cannot be activated by WNTs. We successfully generated the FZD_6_-K466TCO*K–corresponding mutant SMO-E508TCO*K (fig. S6, A to C), confirmed resonance energy transfer in the basal state of the receptor (fig. S6E), and demonstrated, with an absent WNT-induced BRET response in the SMO CRD sensor, that the BRET changes detected with FZD_5_ and FZD_6_ mutants reflect ligand-dependent signals and are not the result of optical interference with the luminescence readout (fig. S6D). Further, we used the same SMO construct and FZD_5_-Q493TCO*K to understand whether the WNT-induced BRET changes in FZDs are due to a change in photophysical properties of the environmentally sensitive BRET acceptor Cy3. We expressed and labeled either of these two sensors in HEK293T cells and measured emission intensity of Cy3 upon external fluorescence excitation in the absence of the Nluc substrate furimazine (fig. S7, A and B). Here, neither WNT-3A (fig. S7C) nor WNT-5A (fig. S7D) induced an FZD_5_-dependent change in Cy3 fluorescence emission, confirming that the WNT-induced BRET signals detected with FZD_5/6_-based, but not SMO-based, biosensors are not due to the environmental sensitivity of Cy3.

The dynamic BRET signal illustrating the conformational rearrangement of the CRD can be induced by WNT-5A in both the FZD_6_-K466K-Cy3 and the FZD_5_-Q493K-Cy3 sensors with similar median effective concentration (EC_50_) values (850.3 ± 84.4 ng/ml and 22.4 ± 2.2 nM for FZD_6_-K466K-Cy3 and 807.1 ± 94.6 ng/ml and 21.2 ± 2.5 nM for FZD_5_-Q493K-Cy3; Fig. 2F). Notably, the potency of WNT-5A measured at FZD_5_-Q493Amb is about fourfold lower compared to the affinity of the same recombinant WNT-5A protein when binding to the purified CRD of FZD_5_ [5.1 ± 1.6 nM; (*9*)]. This difference resembles the affinity-to-potency shift observed previously with conformational GPCR biosensors (*27*). We also tested labeling of the two ECL3 mutant sensors FZD_6_-K466TCO*K and FZD_5_-Q493TCO*K with Tet–BDP-FL and detected WNT-5A–induced BRET responses for both sensors (fig. S8, A to C). In contrast to Cy3-labeled receptors, BDP-FL–labeled sensors show positive BRET amplitudes, which can be explained by changes in relative dipole orientation as observed with other intramolecular GPCR biosensors (*43*, *44*). More specifically, the two negatively charged sulfonate groups in Cy3 will likely be fully hydrated, resulting in high mobility of the chromophore. By contrast, the high lipophilicity of BDP could result in anchoring the chromophore in the lipid bilayer and a confinement of its mobility most likely explaining the opposite BRET changes in response to the same stimulus.

### WNT-3A induced equivalent BRET changes in FZD_5_ and FZD_6_ CRD sensors

While WNT-5A is considered to initiate β-catenin–independent signaling, WNT-3A signals through β-catenin–dependent signaling including phosphorylation of low-density LRP5/6, β-catenin stabilization, and lastly activating the T cell factor (TCF)/lymphoid enhancer factor–dependent gene expression (*9*, *45*) following the principles of the signalosome hypothesis.

**Fig. 3.**
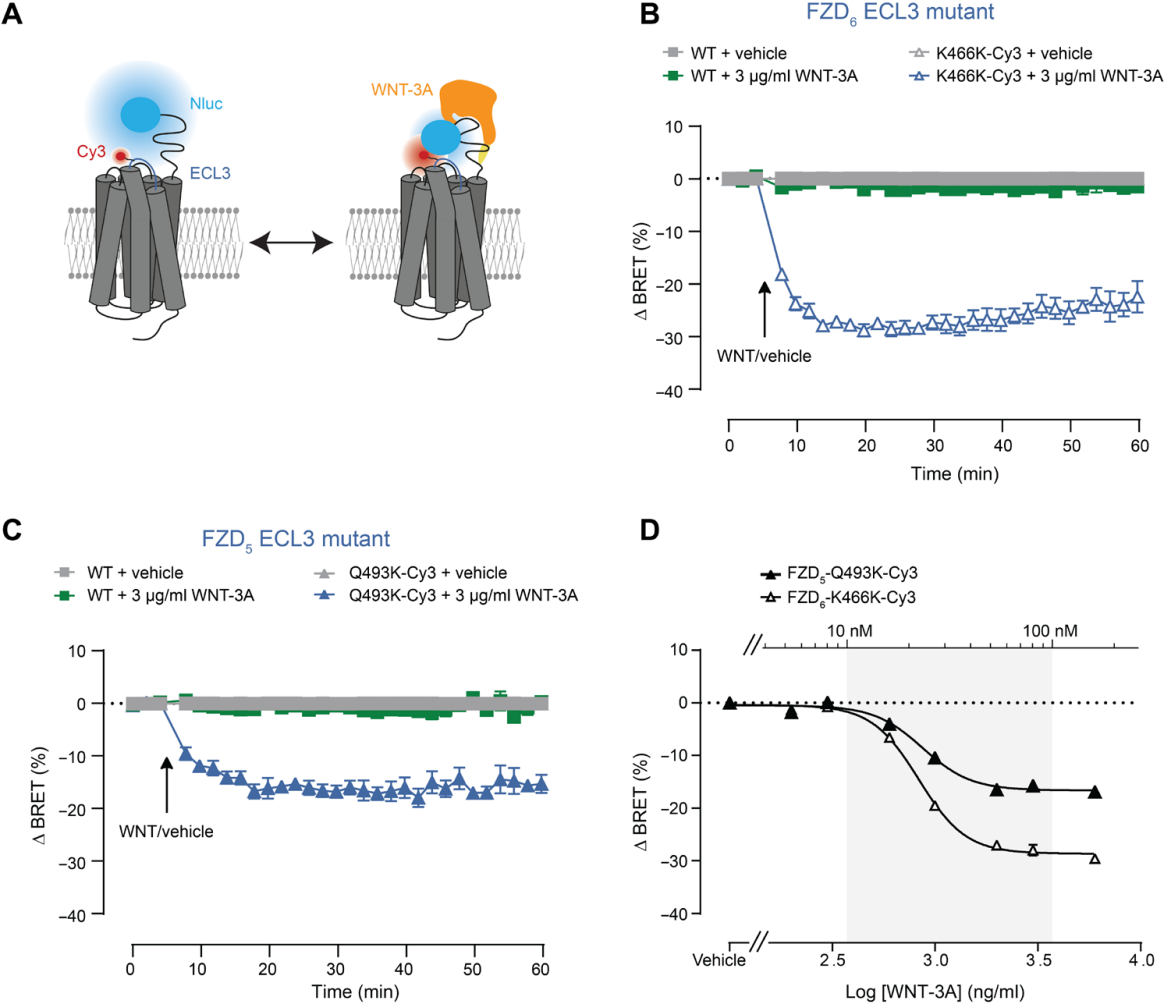
CRD rearrangements in FZD_5_ and FZD_6_ in response to WNT-3A. (**A**) Schematic depiction of the CRD sensor design with the N-terminally Nluc-tagged FZD. Fluorescence labeling of residues in the ECL3 (blue) region of the receptor with Tet-Cy3. (**B** and **C**) BRET responses of FZD_6_ (B) or FZD_5_ (C) ECL3 mutant CRD sensors upon WNT-3A treatment (3 μg/ml) or vehicle control. The arrow indicates the time point of WNT/vehicle application. (**D**) Concentration-response curves of WNT-3A on HEK293T cells expressing FZD_5_-Q493K-Cy3 or FZD_6_-K466K-Cy3. Concentration-response curves are normalized to vehicle control. All experiments were performed in HEK293T cells cotransfected with the indicated FZD_5_ or FZD_6_ sensors and the orthogonal tRNA/synthetase pair.

We aimed to test whether WNT-3A (3 μg/ml), which corresponds to 76 nM (predicted molar mass of WNT-3A according to the supplier: 37,400 Da), is able to induce conformational changes not only in FZD_5_ but also in FZD_6_ known to mediate β-catenin–independent signaling (Fig. 3A). With both sensors, FZD_6_-K466K-Cy3 (Fig. 3B) and FZD_5_-Q493K-Cy3 (Fig. 3C), we were able to detect a WNT-3A–induced BRET response. Equal to WNT-5A, concentration-response curves of WNT-3A applied for 30 min revealed similar EC_50_ values for both receptor sensors (832.8 ± 24.83 ng/ml and 22.3 ± 0.7 nM for FZD_6_-K466 K-Cy3 and 869.6 ± 33.99 ng/ml and 23.3 ± 0.9 nM for FZD_5_-Q493K-Cy3; Fig. 3D). Similar to the results obtained with WNT-5A, the EC_50_ of WNT-3A detected with the Nluc-FZD_5_-Q493K-Cy3 sensor is more than sevenfold higher than the binding affinity of recombinant WNT-3A to the purified CRD of FZD_5_ [3.1 ± 1.0 nM; (*9*)]. This shift is likely not due to impaired ligand binding capacity of the Nluc-tagged FZD_5_ sensor, because BRET-based binding experiments with fluorescently labeled WNT-3A revealed similarly high WNT affinities (compared to the experiments with purified WNTs and CRDs) to Nluc-tagged FZD_5_ and FZD_6_ on the surface of living cells [10.2 ± 3.7 nM for Nluc-FZD_6_ and 2.3 ± 0.2 nM for Nluc-FZD_5_; (*46*)]. Notably, the maximal BRET responses of both the FZD_5_-Q493K-Cy3 and the FZD_6_-K466K-Cy3 sensors, for WNT-3A and WNT-5A, are comparable to each other (table S1) and suggest that WNT-3A could activate FZD_6_ independently of LRP5/6 or, more precisely, induces a conformational change of the CRD upon binding.

### Intramolecular versus intermolecular BRET responses

Little is known about FZD dimerization, but there is evidence that FZD dimerization through a CRD-CRD interaction contributes to WNT-induced β-catenin signaling as it was shown in *Xenopus* FZD_3_ (*47*) or FZD_5_ and FZD_7_ (*48*). FZD_6_ exists as homodimer under basal conditions and undergoes dissociation and reassociation upon WNT stimulation (*49*).

The existence of FZD dimers could result in intermolecular “cross-talk” between Nluc and the FZD_5_ and FZD_6_ sensors of different monomers. To quantify the contribution of intermolecular BRET to the total BRET response of the FZD CRD sensors, we cotransfected an Nluc-tagged FZD WT, which is not per se able to act as a sensor, and an Nluc-lacking FZD amber mutant. While cotransfection of Nluc-FZD_5_-WT and the FZD_5_-Q493Amb mutant did not result in a detectable WNT-induced BRET response in contrast to the BRET response detected with the intramolecular Nluc-FZD_5_-Q493K-Cy3 sensor (fig. S9), cotransfecting Nluc-FZD_6_-WT and the Nluc-lacking FZD_6_-K466Amb mutant sensor did. The WNT-5A–induced BRET response obtained with the intermolecular BRET pair setup, however, was smaller compared to the intramolecular Nluc-FZD_6_-K466K-Cy3 sensor (fig. S10, A and B). Although stoichiometric differences of the BRET partners in these distinct experiments hamper a direct comparison of the ΔBRET amplitudes, the response detected in the intermolecular BRET setup indicated that a minor part of the total BRET change originates from agonist-induced FZD_6_ dimer dissociation. While these findings support the previous data on WNT-induced dimer dissociation (*49*), the small contribution of the intermolecular BRET does not affect the conclusions about the intramolecular BRET changes with regard to CRD rearrangements. The time course data clearly indicated a kinetic difference between the two assay setups, further underlining that distinct mechanistic processes account for the recorded BRET changes. To provide further support of this assumption, we calculated the rate constant *k* by fitting the BRET amplitudes for each of the experimental paradigms with intra- and intermolecular sensors. The higher *k* values for the intramolecular BRET changes compared to the intermolecular sensor argue for a chronological order of the events with faster extracellular conformational changes followed by FZD_6_ dimer dissociation (fig. S10C).

In addition, we made use of the FZD_6_ dimerization–deficient triple Ala mutant D365A/R368A/Y369A (*49*) by introducing three Ala mutations into the Nluc-FZD_6_-K466 amber construct, resulting in the Nluc-FZD_6_-K466TCO*K dimer mutant. The FZD_6_-K466TCO*K dimer mutant maintained cell surface localization even though the surface expression was reduced compared to the FZD_6_-K466TCO*K mutant (fig. S11A), and incubation with Tet-Cy3 showed a distinct fluorescence-labeling capability (fig. S11B). The WNT-5A–induced conformational change resulted in BRET responses showing amplitudes of about half of the FZD_6_-K466K-Cy3 mutant (fitted plateau values: FZD_6_-K466K-Cy3 dimer mutant, −10.22 ± 0.32% and FZD_6_-K466K-Cy3, –19.74 ± 0.68%). Especially in light of the reduced surface expression of the dimer-deficient FZD_6_ sensor, the reduced but yet significant BRET signal argues for dimer dissociation–independent, intramolecular BRET within one monomer between the Nluc fused to FZD’s N terminus and the introduced fluorescent dye in ECL3 (fig. S11C). Furthermore, the basal BRET ratio of the FZD_6_-K466K-Cy3 dimer mutant was significantly reduced compared to the native FZD_6_-K466K-Cy3 sensor, arguing for substantially diminished dimerization tendency of the dimer mutant control (fig. S11D).

Thus, these dimer control experiments indicate that the BRET amplitudes as a consequence of WNT-induced extracellular conformational changes in FZD_6_ present a composite response of both intra- and intermolecular BRET events. In contrast, intermolecular BRET events have no impact on the FZD_5_ CRD sensor, which solely detects intramolecular BRET as a consequence of WNT-induced extracellular conformational changes.

### Kinetic insights into WNT-induced conformational changes of FZDs

**Fig. 4.**
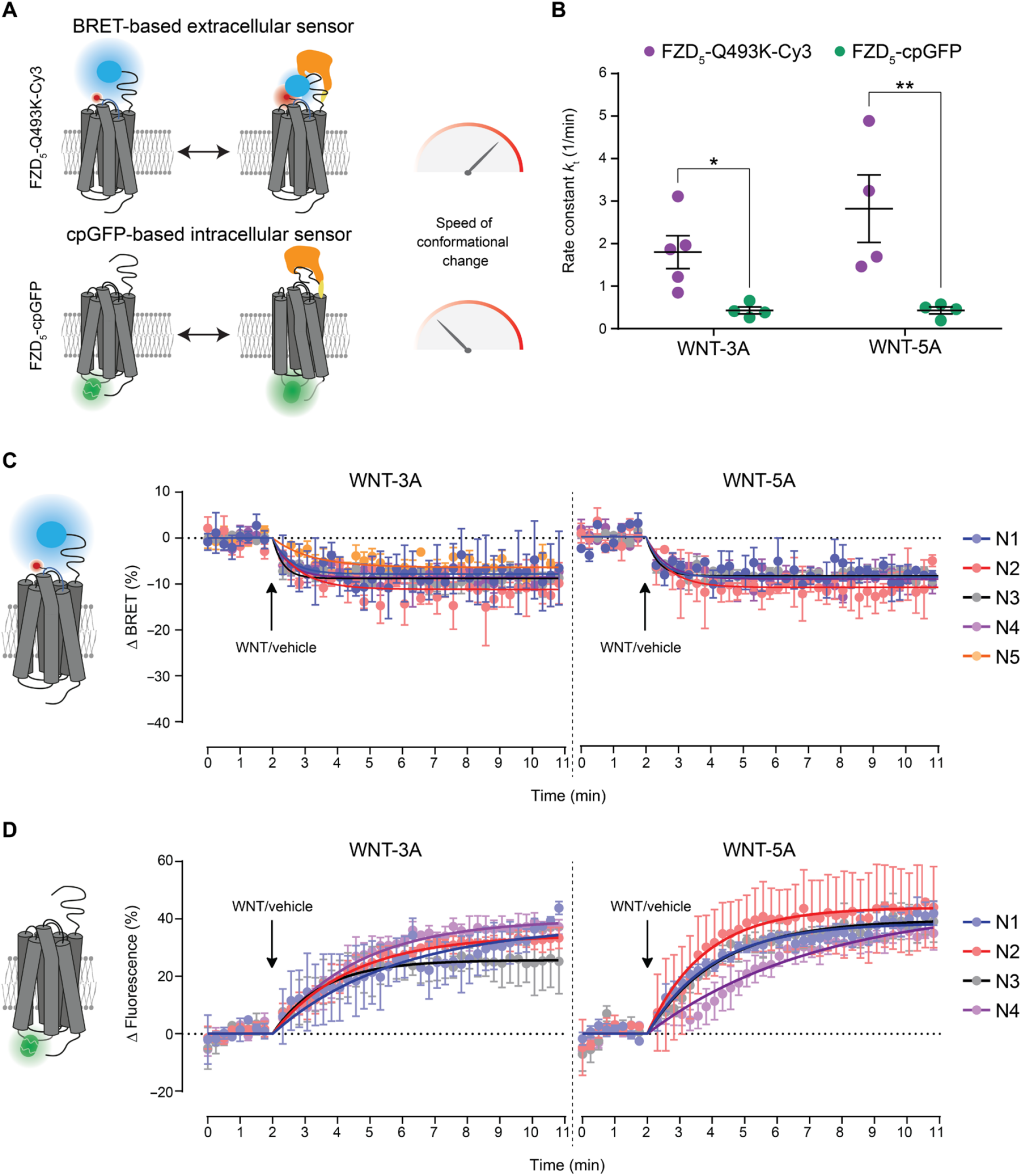
Kinetic insights into WNT-induced conformational changes in FZD_5_. (**A**) Schematic depiction of the CRD sensor design for the BRET-based extracellular sensor FZD_5_-Q493K-Cy3 and the cpGFP-based intracellular sensor FZD_5_-cpGFP upon WNT binding. (**B**) Rate constant *k* of WNT-3A– and WNT-5A–induced BRET responses (FZD_5_-Q493K-Cy3 extracellular CRD sensor) or fluorescence responses (FZD_5_-cpGFP intracellular sensor) determined from fitted data in (C) and (D) using the plateau followed by one-phase decay equation. Differences in WNT-3A– or WNT-5A–induced effects on FZD_5_-Q493K-Cy3 and FZD_5_-cpGFP were analyzed with two-way analysis of variance (ANOVA) followed by Fisher’s least significant difference post hoc test. Significance levels are given as *P < 0.05 and **P < 0.01. (**C**) Kinetic fits of WNT-3A– and WNT-5A–induced BRET responses of HEK293T cells expressing FZD_5_-Q493K-Cy3 of five individual experiments (N1 to N5). (**D**) Kinetic fits of WNT-3A– and WNT-5A–induced fluorescence responses of HEK293A cells stably expressing FZD_5_-cpGFP of four individual experiments (N1 to N4). Data in (C) and (D) show means ± SD of the individual experiments.

WNT-induced FZD dynamics were previously investigated using intracellular fluorescence-based conformational sensors (*20*). We aimed to compare the speed of the WNT-induced conformational rearrangements in distinct domains of FZDs by quantifying and comparing the reaction rates of the extracellular and intracellular conformational FZD sensors (Fig. 4A). Therefore, we stimulated HEK293T cells expressing the FZD_5_-Q493K-Cy3 CRD sensor (Fig. 4, B and C) or HEK293A cells stably expressing the FZD_5_–circularly permutated green fluorescent protein (cpGFP) intracellular sensor (Fig. 4, B to D) with WNT-3A and WNT-5A (3 μg/ml) and recorded the resulting BRET or fluorescence response of the two different sensors over time. The resulting rate constant *k* was found to be significantly higher for the FZD_5_-Q493K-Cy3 CRD sensor for both WNT-3A and WNT-5A (Fig. 4B). The higher rate constant implies that the conformational changes at the extracellular part of FZD occur faster than the intracellular detected rearrangements, highlighting a sequence of events, where WNT-induced conformational changes in the extracellular domain precede those in the core of the receptor.

### The role of LRP5/6 for WNT-induced conformational changes in the CRD

**Fig. 5.**
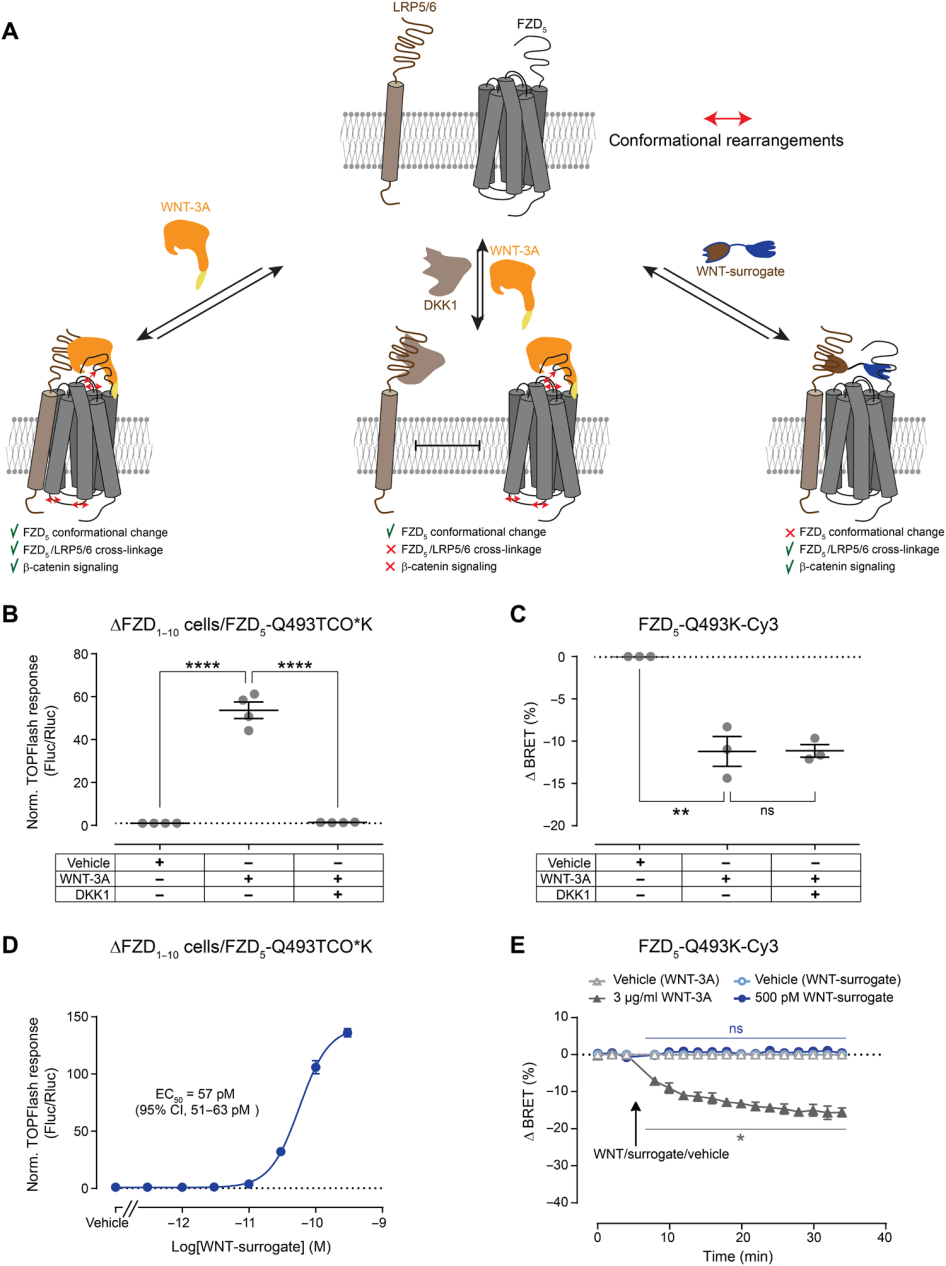
DKK1 and a WNT-surrogate to analyze the role of LRP5/6 for WNT-induced extracellular conformational changes. (**A**) Schematic depiction of the initiation of WNT-induced β-catenin signaling. (**B**) TOPFlash reporter gene response induced by WNT-3A (1 μg/ml) in the absence or presence of DKK1 (1 μg/ml) in ΔFZD_1–10_ HEK293T cells expressing FZD_5_-Q493K-Cy3. Data show means ± SEM of four independent experiments. (**C**) Maximal BRET response induced by WNT-3A (3 μg/ml) in the absence or presence of DKK1 (3 μg/ml) in ΔFZD_1–10_ HEK293T cells expressing FZD_5_-Q493K-Cy3. Results in (B) and (C) were analyzed with one-way ANOVA followed by Tukey post hoc test. Significance levels are given as **P < 0.01 and ****P < 0.0001; ns, not significant. (**D**) TOPFlash reporter gene response induced by increasing concentration of WNT-surrogate in ΔFZD_1–10_ HEK293T cells expressing FZD_5_-Q493K-Cy3. The concentration-response curve of WNT-surrogate is normalized to vehicle control. CI, confidence interval. (**E**) BRET responses of the FZD_5_-Q493K-Cy3 sensor upon WNT-3A (3 μg/ml), 500 pM WNT-surrogate treatment, or vehicle control of four independent experiments. The arrow indicates the time point of WNT/WNT-surrogate/vehicle application. Differences between WNT-surrogate vehicle control and WNT-surrogate or WNT-3A vehicle control and WNT-3A–induced BRET responses were analyzed with multiple *t* test followed by Holm-Sidak multiple comparison. Significance levels are given as *P < 0.05. All experiments were performed in ΔFZD_1–10_ HEK293T cells or HEK293T cells cotransfected with FZD_5_-Q493Amb and the orthogonal tRNA/synthetase pair.

WNT-3A–mediated β-catenin signaling depends on WNT-induced cross-linkage of FZDs with LRP5/6 (Fig. 5A). The cross-linkage can be blocked by the glycoprotein dickkopf-1 (DKK1), known to inhibit β-catenin signaling by binding to the ectodomains of LRP5/6 and thereby preventing WNT engagement with LRP5/6 and subsequent FZD/LRP complex formation in signalosomes (*50*, *51*).

To strengthen the hypothesis that WNTs can promote conformational rearrangements in FZDs independently of co-receptors such as LRP5/6, we preincubated the FZD_5_-Q493K-Cy3 sensor with recombinant DKK1. While DKK1 inhibits WNT-3A–induced FZD_5_/LRP5/6 signaling along the WNT/β-catenin axis (Fig. 5B), DKK1 preincubation did not affect WNT-induced conformational changes of the FZD CRD assessed by robust BRET responses (Fig. 5C), indicating that the WNT-induced conformational change of the CRD did not require FZD/LRP5/6 cross-linking.

In contrast, a surrogate WNT (named as WNT-surrogate), an artificial construct composed of a FZD- and a LRP5/6-binding moiety, initiates β-catenin signaling through a forced cross-linkage of FZD with LRP5/6 (*52*). The WNT-surrogate applied to the FZD_5_-Q493K-Cy3 sensor yielded a concentration-response curve for the induction of a TCF transcriptional response (TOPFlash assay) with an EC_50_ value of 57 pM (95% confidence interval, 51 to 63 pM; Fig. 5D). However, even at a 10× higher concentration (500 pM), the WNT-surrogate was neither able to induce any BRET responses in the FZD_5_ CRD sensor (Fig. 5E) nor fluorescence responses in the cpGFP sensor (fig. S12), arguing for the absence of extracellular and, in addition, intracellular conformational changes. These findings indicate that WNT-3A induces FZD_5_/LRP5/6 cross-linkage and extracellular conformational changes in FZDs, but the latter are not required to initiate β-catenin signaling.

## DISCUSSION

The WNT/FZD system represents a primary component of myriad vital biological processes in human physiology and disease. Binding of WNTs to the CRD of FZDs constitutes the initial step of WNT morphogen signaling (*2*), triggering diverse intracellular signaling cascades through cross-linking of FZDs with WNT co-receptors (most prominently with LRP5/6) (*2*, *13*–*15*) and by inducing intramolecular, conformational dynamics in FZDs independently from co-receptor interaction (*20*). Seminal work in models of *Drosophila melanogaster* has allowed to infer WNT/FZD interaction from intracellular signaling–dependent readouts (*23*, *53*, *54*), and more recent studies with conformational receptor biosensors have provided valuable insights into WNT-induced structural dynamics at the intracellular parts of FZDs (*16*, *18*, *20*). However, how WNT engagement with the extracellular CRD is mechanistically connected to these intracellular receptor conformational changes or FZD/WNT-co-receptor cross-linking remained unclear. The flexibility of the CRD relative to the core of FZDs and its relevance for signal initiation has been a matter of debate, and it remains unclear how the information flow from WNT binding to the CRD is transduced to the core of the receptor provided that the linker domain is freely flexible. The ligand-free CRD of FZD_7_ contributes to constitutive receptor activation toward heterotrimeric G_s_ proteins (*55*). However, the underlying structural aspects of CRD-mediated constitutive activity remain obscure.

Our study provides structural and kinetic insights into this core event of WNT/FZD signaling. We describe the development and validation of optical biosensors that unveil extracellular conformational rearrangements in FZDs in real time in living cells. These biosensors rely on BRET between N-terminally fused Nluc and a fluorescent dye, incorporated in the receptor’s linker region or ECL3, using a minimally invasive technique based on site-directed insertion of uaas and bioorthogonal coupling chemistry in live cells to minimize interference with receptor functionality and ligand/receptor interaction. Although we cannot entirely exclude that sterically induced Nluc displacement triggered by the engagement of a bulky WNT ligand with the CRD contributes to the detected BRET changes, two of our observations argue for a distinct molecular mechanism underlying the optical signals recorded with these biosensors: First, comparing the WNT potencies (EC_50_ values) obtained with these biosensors to previously determined WNT binding affinities reveals a four- to sevenfold shifted concentration-response correlation, similar to what has been described for intramolecular GPCR sensors that report on conformational rearrangements at the cytoplasmic side of these membrane-spanning proteins (*27*). Second, the lack of BRET response seen with the WNT-surrogate ligand, which exhibits an even higher molecular weight than WNT-3A and WNT-5A and is composed of the CRD-binding region of vantictumab, a FZD-targeting antibody blocking WNT-FZD binding (*52*, *56*), implies that sterically induced Nluc relocation cannot be the only trigger of the observed BRET changes. These two observations suggest that the BRET signals revealed by these biosensors reflect global conformational changes occurring in FZDs’ extracellular domains upon WNT binding.

The biosensors present an important step toward understanding the conformational dynamics of the extracellular domains of FZDs, by shedding light on the stepwise processes occurring between WNT-CRD binding and FZD/transducer coupling. Using a set of distinct BRET- and fluorescence-based assays, we show that CRD movements take place before the rearrangement of the receptors’ intracellular domains is initiated and, in the case of FZD_6_, before ligand-induced dissociation of receptor homodimers occurs. Although we cannot provide a causal connection between the two molecular events, our observations indicate that the extracellular CRD rearrangement in FZDs presents an early event underlying signalosome-independent WNT/FZD signaling and confirm—using an unprecedented live cell biosensor system—the central modulatory role of the CRD in class F GPCRs (*33*). It remains to be defined, what molecular movement in fact determines the agonist-induced changes in BRET using the FZD CRD sensors. In an attempt to better understand the consequences of ligand association with the CRD, we overlaid the WNT structure with the different CRD position clusters extracted from the MD simulations. This schematic overlay (fig. S1D) suggests that one mechanism resulting in the detected BRET changes could be the restriction of the range of CRD motion through WNT binding, which could be accompanied by a ligand-induced equilibrium shift toward distinct receptor core conformations. More experiments are required to dissect these structural details of FZD activation.

One important mechanistic finding of our study is that the FZD CRD rearrangements detected by our BRET sensors are not required to initiate β-catenin signaling. Our experiments with a WNT-surrogate known to mediate β-catenin signaling through cross-linkage of FZDs with LRP5/6 (*57*) showed that (i) WNT-surrogate/FZD_5_/LRP5/6 assembly does not provoke conformational changes in the FZD_5_ CRD sensor and (ii) β-catenin–dependent signaling can be mediated by the FZD_5_ biosensor without the type of extracellular conformational changes detected by our FZD CRD sensor. Likewise, blocking WNT-3A–mediated FZD_5_/LRP5/6 assembly with DKK1 had no effect on the CRD dynamics of FZD_5_. These observations support previous notions of a receptor tyrosine kinase–like functionality of FZDs that exclusively relies on clustering FZD and LRP5/6 in a signalosome (*15*, *20*).

Furthermore, we found that WNT-3A and WNT-5A induced very similar BRET responses at saturating concentrations, arguing for analogous CRD rearrangement upon ligand binding. Although we cannot exclude that WNT-3A and WNT-5A promote subtly distinct CRD movements that are not resolved by our FZD CRD sensors, the finding of similar BRET responses is somewhat unexpected in light of the distinct modes of action and intracellular signaling pathways initiated by these two endogenous FZD ligands (FZD/co-receptor cross-linking and β-catenin–dependent signaling by WNT-3A versus FZD conformational changes and β-catenin–independent signaling by WNT-5A). This analogy poses the question of whether WNT-3A, concurrent to co-receptor–dependent signal propagation, mediates similar functionalities of FZD_5/6_ as WNT-5A by inducing conformational changes in the receptor. FZD_5_ alanine mutants of either R^6.32^ or W^7.55^, two residues that stabilize the inactive receptor conformation through π-cation interaction (*8*), are completely deficient in mediating WNT-3A– or WNT-surrogate–induced β-catenin signaling (*15*), arguing for receptor conformational control over WNT-3A–dependent intracellular signal propagation. This also implicates that FZD activation and pathway selectivity are governed by two distinct mechanisms: WNT-mediated signalosome formation feeding into WNT/β-catenin signaling on the one hand and GPCR-like signal propagation triggered by intramolecular conformational changes that link extracellular WNT-FZD interaction to intracellular FZD-transducer coupling on the other. Supporting the hypothesis of two coexisting activation mechanisms in FZDs, WNT-3A mediates phosphorylation of extracellular signal–regulated kinases 1 and 2 (ERK1/2) in primary mouse microglia (*45*) and regulates small guanosine triphosphatase activity in platelets (*58*), processes that are often associated with GPCR/G protein signaling. WNT-3A signaling through the WNT/β-catenin pathway and β-catenin–independent signaling to ERK1/2 occurs simultaneously in mouse primary microglia, albeit with different kinetics, regulating the proinflammatory status of these brain macrophages. While the β-catenin–dependent signaling pathway is sensitive to DKK1 treatment, phosphorylation of ERK1/2 is not affected by DKK1-mediated inhibition of FZD-LRP cross-linkage (*45*). In line with these previous findings, DKK1 pretreatment affects neither the WNT-induced BRET response in FZD CRD nor the fluorescence response in the FZD-cpGFP sensors (*20*). In summary, these results underline that FZDs can respond to WNTs with signalosome-dependent and signalosome-independent, FZD conformation–dependent signal initiation, which provides a molecular and mechanistic basis for FZD functional selectivity.

In summary, the sensor design described here presents a universal approach to develop cell-based optical probes for membrane-spanning proteins, including other classes of GPCRs, receptor tyrosine kinases, or cytokine receptors, aiding in the mechanistic exploration of fundamental biological processes underlying ligand initiation, kinetics of receptor conformational changes, and cellular signaling in general.

## MATERIALS AND METHODS

### Experimental design

The objectives of this study were to (i) develop a generalizable biosensor design that allows the assessment of the extracellular conformational dynamics of FZDs upon stimulation with WNTs and to (ii) use these sensors to explore the mechanistic details underlying WNT-FZD signaling in living cells. The conformational FZD sensors were expressed in parental and ΔFZD_1–10_ HEK293T cells and stimulated with recombinant, commercially available WNTs in at least three independent experiments. The WNT-induced changes in FZD conformation and signaling activity were recorded in 96-well microtiter plates and corrected for vehicle responses.

### Computational studies

The FZD_6_ model (amino acids 22 to 511) was built using iTasser server (https://zhanggroup.org/I-TASSER/) (*39*). The protocol selected 10 templates for the modeling: 2ZIY (squid rhodopsin), 4JKV (inactive ΔCRD-SMO), 4ZWJ (rhodopsin-arrestin complex), 5L7D (inactive SMO), 5NDD (protease-activated receptor-2), 6BD4 (ΔCRD-FZD_4_), 6FJ3 (parathyroid hormone 1 receptor), 6LW5 (formyl peptide receptor 2), 6ME6 (melatonin receptor MT2), and 6WW2 (ΔCRD-FZD_5_). After visual examination of the produced five models, two models—models 1 and 3, which both were close to our previous inactive ΔCRD-FZD_6_ model (*20*) [root mean square deviation (RMSD) of 2.4 and 3.2 Å, respectively] and had similarly folded CRDs than FZD_5_ (PDB ID: 5URZ) and FZD_7_ (PDB ID: 5T44)—were picked for a preliminary MD study (ca. 150 ns of production simulation after the equilibration protocol; for details, see below). Only model 1 remained stable in the preliminary simulation and was thus selected for the further MD study.

The MD simulations were run using GROMACS 2020.3 (*59*). FZD_6_ was oriented by aligning it to the FZD_4_ from the Orientations of Proteins in Membranes (OPM) database database (https://opm.phar.umich.edu/) and embedded in the phosphatidylcholine (POPC) lipid bilayer (150 lipids per leaflet) by CHARMM-GUI server (www.charmm-gui.org/) with TIP3p water molecules and 0.15 M NaCl. The system was minimized for approximately 2000 steps and then equilibrated with gradually decreasing position restraints on protein and lipid components. In the last 50 ns of the equilibration run, the harmonic force constants of 50 kJ mol^−1^ nm^−2^ were applied on the protein atoms only.

Seven (250 ns) independent isobaric and isothermic (NPT) ensemble production simulations were initiated using the CHARMM36m force field (*60*) and a 2-fs time step. First, replica 1 was run starting from the equilibrated structure and random velocities. Then, six other replicas were simulated starting from the snapshots of replica 1 at time points *t* = 0 ns, *t* = 50 ns, *t* = 100 ns, *t* = 150 ns, *t* = 200 ns, and *t* = 250 ns and random velocities. The temperature at 303.15 K was maintained with a Nose-Hoover thermostat (*61*), and the pressure at 1 bar was maintained with a Parrinello-Rahman barostat (*62*). Potential-shift-Verlet was used for electrostatic and van der Waals interactions with a 12-Å cutoff, and the bonds between hydrogen and other atoms were constrained by the Linear Constraint Solver for Molecular Simulations (LINCS) algorithm (*63*). The data were analyzed using VMD (visualization, measurement of RMSDs and distances, and RMSD clustering) (*64*) and visualized in PyMol. For RMSD clustering, the size of the trajectory was reduced to contain one frame per every 10 ns of the simulation, the frames were superimposed on the 7TM core of the receptor at the first frame of the first replica, and similarity cutoff was set to 5 Å and maximum number of clusters to 30. The cluster seeds were used as the representative models of each cluster. The MD data will be deposited to GPCRmd (an open-access MD database for GPCRs; www.gpcrmd.org).

### Cloning of FZD constructs

The synthetic gene constructs for FZD_5_ and FZD_6_ were designed on the basis of the amino acid sequences of the full-length receptor lacking the native signal peptide [residues 27 to 585 of Uniprot Q13467 (FZD5_HUMAN) and residues 19 to 706 of Uniprot O60353 (FZD6_HUMAN)] and codon-optimized for expression in human cells using the GeneArt online tool while avoiding a set of motifs corresponding to several restriction sites (Nhe I, Hind III, Nco I, Eco RI, Sbf I, Mfe I, Kpn I, Not I, and Xba I). We extended the 5′ end of the genes with the nucleotide sequence 5′-AAG CTT GCC GCC ACC ATG GCG CTG TGT ATC CCT CAA GTT CTG CTG GCC CTG TTC CTG AGC ATG CTG ACA GGA CCT GGC GAG GGC TAC CCT TAC GAT GTG CCT GAC TAC GCC GAA TTC GCT CCT GCA GGG AGT CAA TTG-3′ that adds the restriction site Hind III used for cloning and the protein sequence MALCIPQVLLALFLSMLTGPGEGYPYDVPDYAEFAPAGSQL to the N terminus of the receptor. This sequence is derived from the mouse serotonin 5-HT_3_ receptor–cleavable signal sequence carrying the R2A mutation to enable usage of a strong Kozak consensus sequence (GCCGCCACCATGG, start codon underlined) and a hemagglutinin tag (YPYDVPDYA) followed by a linker EFAPAGSQL corresponding to a nucleotide sequence with Eco RI, Sbf I, Mfe I, and Mly I sites (*65*). We extended the 3′ end with the sequence 5′-GGT ACC GCC TCC TCG GAT GAG GCC AGC ACA ACC GTG TCT AAG ACC GAG ACA TCT CAG GTG GCC CCT GCC TAA GCG GCC GCT CTA GA-3′ containing a Kpn I site at the 5′ end and Not I and Xba I sites at the 3′ end. The C-terminal 18-residue-long sequence is the rhodopsin 1D4 mAb epitope tag (*66*). The constructs were synthesized and cloned into a plasmid derived from pcDNA3.1(+) modified to eliminate an internal Mfe I site.

All FZD_5_ and FZD_6_ amber mutants were generated using the GeneArt Site-directed Mutagenesis System (Thermo Fisher Scientific) with the following primers: FZD_6_-Q171Amb, 5′-AAA ACA TCT GGC GGC TAG GGC TAC AAG TTC C-3′ (forward) and 5′-GGA ACT TGT AGC CCT AGC CGC CAG ATG TTT T-3′ (reverse); FZD_6_-K174Amb, 5′-GGC GGC CAG GGC TAC TAG TTC CTG GGC ATC G-3′ (forward) and 5′-CGA TGC CCA GGA ACT AGT AGC CCT GGC CGC C-3′ (reverse); FZD_6_-D179Amb, 5′-AAG TTC CTG GGC ATC TAG CAG TGC GCC CCT CCA-3′ (forward) and 5′-TGG AGG GGC GCA CTG CTA GAT GCC CAG GAA CTT-3′ (reverse); FZD_6_-Q180Amb, 5′-TTC CTG GGC ATC GAT TAG TGC GCC CCT CCA T-3′ (forward) and 5′-ATG GAG GGG CGC ACT AAT CGA TGC CCA GGA A-3′ (reverse); FZD_6_-V450Amb, 5′-TGG GAG ATC ACA TGG TAG TCC GAC CAC TGC AG-3′ (forward) and 5′-CTG CAG TGG TCG GAC TAC CAT GTG ATC TCC CA-3′ (reverse); FZD_6_-K466Amb, 5′-TGT CCA TAC CAG GCC TAG GCC AAA GCC AGA C-3′ (forward) and 5′-GTC TGG CTT TGG CCT AGG CCT GGT ATG GAC A-3′ (reverse); FZD_6_-K468Amb, 5′-TAC CAG GCC AAG GCC TAG GCC AGA CCT GAG CTG-3′ (forward) and 5′-CAG CTC AGG TCT GGC CTA GGC CTT GGC CTG GTA-3′ (reverse); FZD_5_-Q493Amb, 5′-GGC CAC GAT ACA GGC TAG CCT AGA GCC AAG C-3′ (forward) and 5′-GCT TGG CTC TAG GCT AGC CTG TAT CGT GGC C-3′ (reverse).

To boost the amber suppression, we introduced the respective amber mutant-bearing FZD into an expression plasmid carrying four repeats of the orthogonal suppressor tRNA (plasmid as a gift from S. Elsässer, Addgene number: 140008).

For cloning the BRET extracellular sensors, an Nluc tag in combination with FZD_5_ or FZD_6_ was subcloned into the tRNA expression vector using the NEBuilder HiFi DNA Assembly Kit (New England Biolabs). Briefly, the tRNA expression vector was digested with Nhe I and Bam HI. The Nluc tag was obtained from the Nluc-FZD_6_ construct (*16*) and contains the N-terminal protein sequence MRLCIPQVLLALFLSMLTGPGEGSRKL (signal peptide derived from the 5-HT_3_ receptor, followed by the Hind III restriction site and Nluc). The Nluc tag was cloned N-terminally of FZD_5_ or FZD_6_, both connected via a linker (EFAPAGSQL, corresponding to a nucleotide sequence with Eco RI, Sbf I, Mfe I, and Mly I), with the following primers: Nluc, 5′-TCC AAG CTG TGA CCG GCG CCT ACT CTA GAG CTA GCC ACC ATG CGG CTC TGC-3′ (forward) and 5′-TGC AGG AGC GAA TTC CGC CAG AAT GCG TTC GCA C-3′ (reverse); FZD_5/6_, 5′-GAA CGC ATT CTG GCG GAA TTC GCT CCT GCA GGG AGT C-3′ (forward) and 5′-GCA GAC AGC GAA TTA ATT CCA GCG GCC GCG GAT CCG GCC GCT TAG GCA GGG GC-3′ (reverse). For the dimer control construct, FZD_5_-Q493Amb or FZD_6_-K466Amb was subcloned without the N-terminally Nluc tag into the tRNA expression vector using the NEBuilder HiFi DNA Assembly Kit with the following primers: FZD_5/6_, 5′-GCT GTG ACC GGC GCC TAC TCT AGA GCT AGC GCC GCC ACC ATG GCG CTG-3′ (forward) and 5′-CAG CGA ATT AAT TCC AGC GGC CGC GGA TCC GGC CGC TTA GGC AGG GGC-3′ (reverse). The tRNA expression vector was digested with Nhe I and Bam HI. The triple alanine mutant D365A/R368A/Y369A mutant (called the FZD_6_-K466Amb/TCO*K/K-Cy3 dimer mutant) was cloned using the GeneArt Site-Directed Mutagenesis PLUS Kit (Thermo Fisher Scientific) into the tRNA-Nluc-FZD_6_-K466Amb plasmid with the following primers: D365A/R368A/Y369A, 5′-GCC TGT ACG ATC TGG CCG CCA GCG CGG CCT TTG TGC TCC TGC C-3′ (forward) and 5′-GGC AGG AGC ACA AAG GCC GCG CTG GCG GCC AGA TCG TAC AGG C-3′ (reverse).

For cloning the Nluc-SMO construct, coding for mouse SMO, a 1D4 tag was introduced upstream of the stop codon of the Nluc-SMO plasmid (*67*). The 1D4 tag was introduced in a two-step site-directed mutagenesis process using the GeneArt Site-Directed Mutagenesis PLUS Kit (Thermo Fisher Scientific) with the following primers: SMO-1D4–first, 5′-GCA G**A**C TCG GAC TTC ACC GAG ACA TCT TGA TCT AGA GGG CCC-3′ (forward) and 5′-GGG CCC TCT AGA TCA AGA TGT CTC GGT GAA GTC CGA GTC TGC-3′ (reverse); SMO-1D4–second, 5′-AGA CTC GGA CTT CAC CGA GAC ATC TCA GGT GGC CCC TGC CTG ATC TAG AGG GCC CGT TTA AAC CC-3′ (forward) and 5′-GGG TTT AAA CGG GCC CTC TAG ATC AGG CAG GGG CCA CCT GAG ATG TCT CGG TGA AGT CCG AGT CT-3′ (reverse). Subsequently, an amber codon was introduced at the position E508 using the GeneArt Site-directed Mutagenesis System (Thermo Fisher Scientific) with the following primers: SMO-E508Amb, 5′-CCC ATT CCT GAC TGT TAG ATC AAG AAT CGG C-3′ (forward) and 5′-GCC GAT TCT TGA TCT AAC AGT CAG GAA TGG G-3′ (reverse). Last, the Nluc-mSMO-E508Amb-1D4 was subcloned into the tRNA expression vector as described earlier in this section using the following primers: Nluc-SMO-E508Amb-1D4, 5′-TCC AAG CTG TGA CCG GCG CCT ACT CTA GAG CTA GCC ACC ATG CGG CTC TGC-3′ (forward) and 5′-GCA GAC AGC GAA TTA ATT CCA GCG GCC GCG GAT CCT CAG GCA GGG GCC ACC TG-3′ (reverse). All cloned constructs were verified by sequencing (Eurofins Genomics).

### Cell culture, transfection, and treatments

HEK293T cells cultured in Dulbecco’s modified Eagle’s medium (DMEM) with 1% penicillin/streptomycin and 10% fetal bovine serum (all from Thermo Fisher Scientific) in a humidified 5% CO_2_ incubator at 37°C. Cells were transfected 24 hours after seeding with Lipofectamine 2000 according to the supplier’s information (Invitrogen). The absence of mycoplasma contamination was routinely confirmed by polymerase chain reaction using 5′-GGC GAA TGG GTG AGT AAC ACG-3′ (forward) and 5′-CGG ATA ACG CTT GCG ACT ATG-3′ (reverse) primers detecting 16*S* ribosomal RNA of mycoplasma in the media after 2 to 3 days of cell exposure.

Treatments were done 48 hours after transfection using the following agents: recombinant WNT-3A (R&D Systems, 5036-WN-010), recombinant WNT-5A (R&D Systems, 645-WN-010), WNT-surrogate-Fc fusion protein [U-Protein Express B.V., N001; (*57*)], and DKK1 (R&D Systems, 5439-DK-010/CF). The lyophilized preparations of recombinant WNTs and DKK1 were dissolved in 0.1% bovine serum albumin (BSA)/Dulbecco’s phosphate-buffered saline (DPBS) and stored at 4° to 8°C for a maximum of 4 weeks. Lyophilized WNT-surrogate was dissolved in 0.1% BSA/DPBS and stored at 4° to 8°C for a maximum of 4 weeks. Prior treatment in cell-based experiments, 10-fold compound dilutions were prepared in Sigmacote (Merck)–precoated transparent 96-well plates to avoid adsorption of recombinant proteins to plastic surfaces. All pipetting steps were performed with Sigmacote-precoated pipette tips.

### Immunoblotting

The day prior transfection, 100,000 HEK293T cells per well were seeded in 24-well plates. The cells were transfected with a defined transfection ratio of 9:1 with 0.45 μg of the indicated constructs and 0.05 μg of tRNA/synthetase (Addgene number: 140023; control conditions were balanced with pcDNA) per well and were cultured in the absence or presence of 0.1 mM TCO*K (Sichem, SC-8008). Because FZD_5_-Q493TCO*K was weakly expressed, 300,000 HEK293T cells per well were seeded in 12-well plates. The cells were transfected with 0.9 μg of the FZD_5_-Q493Amb construct or 0.18 μg of FZD_5_-WT/0.72 μg of pcDNA3.1 and 0.1 μg of tRNA/synthetase (control conditions were balanced with pcDNA). Cells were lysed 48 hours after transfection in 2× Laemmli buffer containing 200 mM dithiothreitol (Merck). Lysates were sonicated and separated by SDS–polyacrylamide gel electrophoresis/immunoblotting using 7.5% gels. Transfer to a polyvinylidene difluoride membrane was done with the Trans-Blot Turbo Transfer System (Bio-Rad). After transfer, membranes were incubated in 5% low-fat milk/TBS-T [25 mM tris-HCl, 150 mM NaCl, and 0.05% Tween 20 (pH 7.6)] and subsequently in primary antibodies overnight at 4°C. The next day, the membranes were washed four times in TBS-T, incubated with goat anti-mouse or goat anti-rabbit secondary antibody conjugated to horseradish peroxidase (Thermo Fisher Scientific, 1:5,000), washed, and developed using Clarity Western ECL Substrate (Bio-Rad) according to the supplier’s information. Primary antibodies were as follows: anti-1D4 (National Cell Culture Center, mouse; 1:1000), anti-Nluc (R&D Systems, MAB100261-SP, mouse; 1:500), and glyceraldehyde-3-phosphate dehydrogenase (Cell Signaling Technology, 2118, rabbit, 1:4000).

### Whole-cell ELISA

For quantification of cell surface receptor expression, 15,000 HEK293T cells were plated in 96-well plates precoated with poly-d-lysine (PDL). Next day, cells were transfected with 0.09 μg of the indicated constructs and 0.01 μg of tRNA/synthetase and were cultured in the absence or presence of 0.1 mM TCO*K. After 48 hours, cells were incubated with an anti-Nluc antibody (R&D Systems, MAB100261-SP, mouse; 1:500) in 1% BSA/DPBS for 1 hour at 4°C. Following incubation, cells were washed five times with 0.5% BSA/DPBS and probed with a horseradish peroxidase–conjugated goat anti-mouse antibody at a 1:2500 dilution in 1% BSA/DPBS for 1 hour at 4°C. The cells were washed five times with 0.5% BSA/DPBS, and 100 μl of the peroxidase substrate 3,3′,5,5′-tetramethylbenzidine (Merck) was added (30 min at room temperature). After acidification with 100 μl of 2 M HCl, the absorbance was read at 450 nm using a POLARstar Omega plate reader (BMG Labtech).

### Live cell imaging

The day prior transfection, 15,000 HEK293T cells per well were seeded into PDL-precoated black 96-well glass bottom plates. The cells were transfected with 0.09 μg of the indicated constructs and 0.01 μg of tRNA/synthetase and were cultured in the absence or presence of 0.1 mM TCO*K. Forty-eight hours after transfection, cells were washed in DPBS, kept for 2 hours in DMEM to remove remaining TCO*K, and labeled with 1 μM Tet-Cy3 (Jena Bioscience, CLK-014-05) or Tet–BDP-FL (Jena Bioscience, CLK-036-05) for 30 min. Cells were washed again with DPBS and kept for an additional 30 min in DMEM. Last, DMEM was exchanged with 0.1% BSA/Hanks’ balanced salt solution (HBSS), and cells were imaged using a Zeiss LSM880 confocal microscope.

### Assessment of fluorescence labeling efficacy

For quantification of the fluorescence labeling of the receptor mutants, 15,000 HEK293T cells were plated in black PDL-precoated 96-well plates. Next day, cells were transfected with 0.09 μg of the indicated constructs and 0.01 μg of tRNA/synthetase and were cultured in the absence or presence of 0.1 mM TCO*K. Forty-eight hours after transfection, cells were washed in DPBS, kept for 2 hours in DMEM, and labeled with 1 μM Tet-Cy3 (Jena Bioscience, CLK-014-05) or Tet–BDP-FL (Jena Bioscience, CLK-036-05) for 30 min. Cells were washed again with DPBS and kept for an additional 30 min in DMEM. Last, DMEM was exchanged with 0.1% BSA/HBSS, and fluorescence intensities were read using a POLARstar Omega plate reader (BMG Labtech, Ortenberg, Germany) equipped with filters for Cy3 (excitation, 544 nm/emission, 590 nm) and BDP-FL (excitation, 485 nm/emission, 520 nm).

### Nluc-FZD BRET measurements

For BRET measurements with the Nluc-FZD_5/6_ CRD sensors, 15,000 HEK293T cells were plated in PDL-precoated white 96-well plates. Next day, cells were transfected with 0.09 μg of the indicated constructs and 0.01 μg of tRNA/synthetase and were cultured in the presence of 0.1 mM TCO*K. Forty-eight hours after transfection, cells were washed in DPBS, kept for 2 hours in DMEM, and labeled with 1 μM Tet-Cy3 or Tet–BDP-FL for 30 min. Cells were washed with DPBS and kept for an additional 30 min in DMEM. Next, cells were again washed with DPBS and incubated with 90 μl of a 1/1000 dilution of furimazine stock solution (Promega) in 0.1% BSA/HBSS. After 5 min of incubation, the basal BRET ratio was measured in three consecutive reads, and 10 μl of a WNT-3A or WNT-5A solution (3 μg/ml; in 0.1% BSA/HBSS) or vehicle control was applied per well. Subsequently, the BRET ratio was recorded for an additional 25 to 60 min. For experiments with a higher temporal resolution, eight baseline BRET reads were recorded within 2 min prior manual addition of compounds or vehicle control, followed by at least 40 reads. All experiments were conducted using a CLARIOstar plate reader (BMG Labtech, Ortenberg, Germany) equipped with monochromators to separate Nluc (450/80 nm), BDP-FL (520/40 nm), and Cy3 (580/30 nm), respectively.

### Nluc-FZD and Nluc-SMO fluorescence measurements

For fluorescence measurements with the Nluc-FZD_5_-Q493K-Cy3 and Nluc-SMO-E508K-Cy3 CRD sensors, 15,000 HEK293T cells were plated in PDL-precoated black 96-well plates. Next day, cells were transfected with 0.09 μg of the indicated constructs and 0.01 μg of tRNA/synthetase and were cultured in the presence of 0.1 mM TCO*K. Forty-eight hours after transfection, cells were washed in DPBS, kept for 2 hours in DMEM, and labeled with 1 μM Tet-Cy3 for 30 min. Cells were washed with DPBS and kept for an additional 30 min in DMEM. Next, cells were again washed with DPBS and incubated with 90 μl of 0.1% BSA/HBSS. Baseline fluorescence was recorded in three consecutive reads, followed by application of 10 μl of a WNT-3A or WNT-5A solution (3 mg/ml; in 0.1% BSA/HBSS) or vehicle control per well, and the resulting fluorescence intensity was recorded for an additional 45 min using a CLARIOstar plate reader (BMG Labtech, Ortenberg, Germany) equipped with filters to excite Cy3 (580/30 nm).

### FZD-cpGFP experiments

HEK293A cells stably expressing FZD_5_-cpGFP (*20*) were seeded at a density of 80,000 cells per well onto PDL-precoated, black-wall, black-bottomed 96-well plates. Twenty-four hours later, all wells were washed with HBSS and incubated with 0.1% BSA/HBSS. Baseline fluorescence was recorded in eight consecutive reads within 2 min, 10 μl of 10-fold WNT solution or vehicle control was applied per well, and the resulting fluorescence intensity was recorded for an additional 40 reads. All experiments were conducted using a CLARIOstar plate reader (BMG Labtech, Ortenberg, Germany) equipped with filters to excite cpGFP (470/15 nm) and record its emission intensity (515/20 nm). Forty flashes were applied per data point.

### TOPFlash reporter gene assay

ΔFZD_1–10_ HEK293T cells (400,000 cells/ml) were transfected in suspension with 400 ng of M50 Super 8xTOPFlash, 100 ng of pRL-TK Luc, 450 ng of Nluc-FZD_5_-Q493Amb, and 50 ng of tRNA/synthetase per milliliter of cell suspension; supplemented with 0.1 mM TCO*K; and seeded onto PDL-precoated white-wall, white-bottomed 96-well plates (50,000 cells per well). Twenty-four hours after transfection, cells were washed with 100 μl of HBSS and incubated for 4 hours in fetal bovine serum–reduced (0.5%) DMEM (72 μl per well) supplemented with 10 nM C59. Thereafter, 8 μl of recombinant WNT-3A (10 μg/ml) and/or DKK1 (in 0.1% BSA/HBSS) and varying concentrations of WNT-surrogate or the respective vehicle controls were added. Twenty-four hours after stimulation, cells were washed with HBSS and lysed in 30 μl of Promega’s dual luciferase passive lysis buffer. Subsequently, 20 μl of luciferase assay reagent (LARII) was added to each well, and β-catenin–dependent firefly luciferase (Fluc) intensity was measured using a CLARIOstar microplate reader (580/80 nm; 1-s integration time). Next, 20 μl of Stop&Glo Reagent was added to quantify *Renilla* luciferase (Rluc) emission intensity (480/80 nm; 1-s integration time) to control for variations in cell number and transfection efficiency.

### Statistical analysis

All immunoblot experiments are representative of three independent experiments. Statistical and graphical analysis was done using GraphPad Prism 9 software. For analyzing the surface expression, the basal absorbance detected in pcDNA-transfected HEK293 cells was subtracted from all data, and mean values were normalized to WT FZD_5_ or FZD_6_, which was set to 100%. Differences among the TCO*K-untreated and TCO*K-treated groups were analyzed by one-way analysis of variance (ANOVA) with uncorrected Fisher’s least significant difference (LSD) test. Significance levels are given as follows: *P < 0.05, **P < 0.0196, ***P < 0.001, and ****P < 0.0001. All data points represent normalized values, each performed in triplicate. Bars show means ± SEM of three to four independent experiments.

Differences in fluorescence labeling among the TCO*K-untreated and TCO*K-treated groups were analyzed by one-way ANOVA with uncorrected Fisher’s LSD test. Significance levels are given as follows: *P < 0.05, **P < 0.01, ***P < 0.001, and ****P < 0.0001. All data points represent mean values, each performed in triplicate. Bars show means ± SEM of three to six independent experiments.

BRET ratios were interpreted as acceptor emission/donor emission. At least three individual BRET reads were averaged before ligand/vehicle application. For quantification of ligand-induced changes, ΔBRET was calculated for each well as a percentage over basal BRET. Subsequently, the average ΔBRET of the vehicle control was subtracted. All data points represent mean values, each performed in triplicate or quadruplicate, ± SEM of three to five independent experiments. For analyzing the ΔBRET data, the WNT-induced averaged BRET responses were fitted with a plateau followed by one-phase decay equation. Data from concentration-response experiments were fitted using a four-parameter fit. All data are represented as means ± SEM of at least three independent experiments. Data from TOPFlash experiments were expressed as ratios of Fluc over Rluc luminescence intensity to correct for distinct transfection efficiencies in the different samples. The resulting TOPFlash ratios were subsequently normalized for the average TOPFlash ratio of vehicle-treated wells to express WNT-induced changes as increases over baseline.
