# Supplementary material for: Frizzled BRET sensors based on bioorthogonal labeling of unnatural amino acids reveal WNT-induced dynamics of the cysteine-rich domain
Source: Sci Adv. 2021 Nov 10;7(46):eabj7917. doi: 10.1126/sciadv.abj7917 (PMC8580317; doi:10.1126/sciadv.abj7917)
Supplement: Supplementary file 1 — Figs. S1 to S12 Table S1 [file sciadv.abj7917_sm.pdf]

Science Advances
AAAS

Supplementary Materials for

Frizzled BRET sensors based on bioorthogonal labeling of unnatural amino acids reveal WNT-induced dynamics of the cysteine-rich domain

Maria Kowalski-Jahn, Hannes Schihada, Ainoleena Turku, Thomas Huber, Thomas P. Sakmar, Gunnar Schulte*

*Corresponding author. Email: gunnar.schulte@ki.se

Published 10 November 2021, *Sci. Adv.* **7**, eabj7917 (2021)
DOI: 10.1126/sciadv.abj7917

This PDF file includes:

Figs. S1 to S12
Table S1

Fig. S1

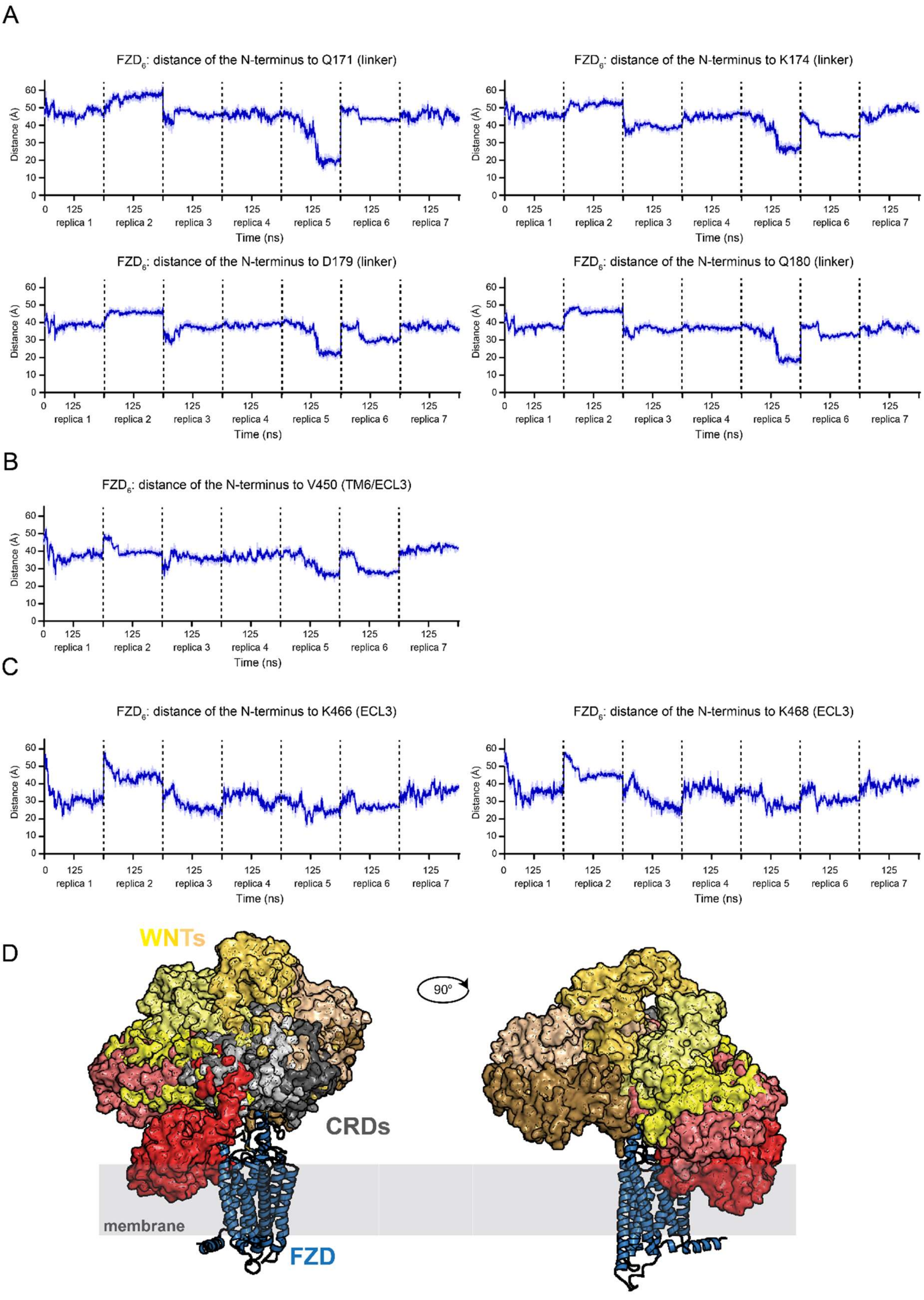
A
FZD6: distance of the N-terminus to Q171 (linker)
FZD6: distance of the N-terminus to K174 (linker)
FZD6: distance of the N-terminus to D179 (linker)
FZD6: distance of the N-terminus to Q180 (linker)
Distance (Å)
Time (ns)
replica 1
replica 2
replica 3
replica 4
replica 5
replica 6
replica 7
B
FZD6: distance of the N-terminus to V450 (TM6/ECL3)
C
FZD6: distance of the N-terminus to K466 (ECL3)
FZD6: distance of the N-terminus to K468 (ECL3)
D
WNTs
CRDs
membrane
FZD
90°

Fig. S1. Distances between the unnatural amino acid (uaa) mutants and the N-terminus of the FZD_6 model throughout the simulation trajectory.

(**A**) Mutants located at the disulfide bridge-stabilized linker region. (**B**) Mutants located at the extracellular extension of TM6. (**C**) Mutants located at the ECL3. The distances in (**A**) to (**C**) are measured between the β-carbons of the residues picked for the point mutation and the N-terminal nitrogen atom and plotted as a continuous trajectory. Dotted lines mark the independent simulation replicas, thick blue traces indicate the moving average smoothed over a 2 ns window and thin traces the raw data. (**D**) Schematic and hypothetical presentation of WNT binding to seven CRD clusters obtained from the MD simulations. The model of xWNT-8 from the xWNT-8-mFZD8-CRD structure (PDB ID: 4F0A) was superimposed to CRDs of seven FZD_6 clusters. One FZD_6 core is colored in blue, CRDs of the different FZD_6 cluster are colored in different shades of grey, WNTs are colored in shades of yellow or red depending on its position relative to the cell membrane (= red means WNT clashes with cell membrane). Approximate localization of the plasma membrane is indicated with grey shading. CRD, cysteine-rich domain; ECL3, extracellular loop 3; TM, transmembrane domain.

Fig. S2

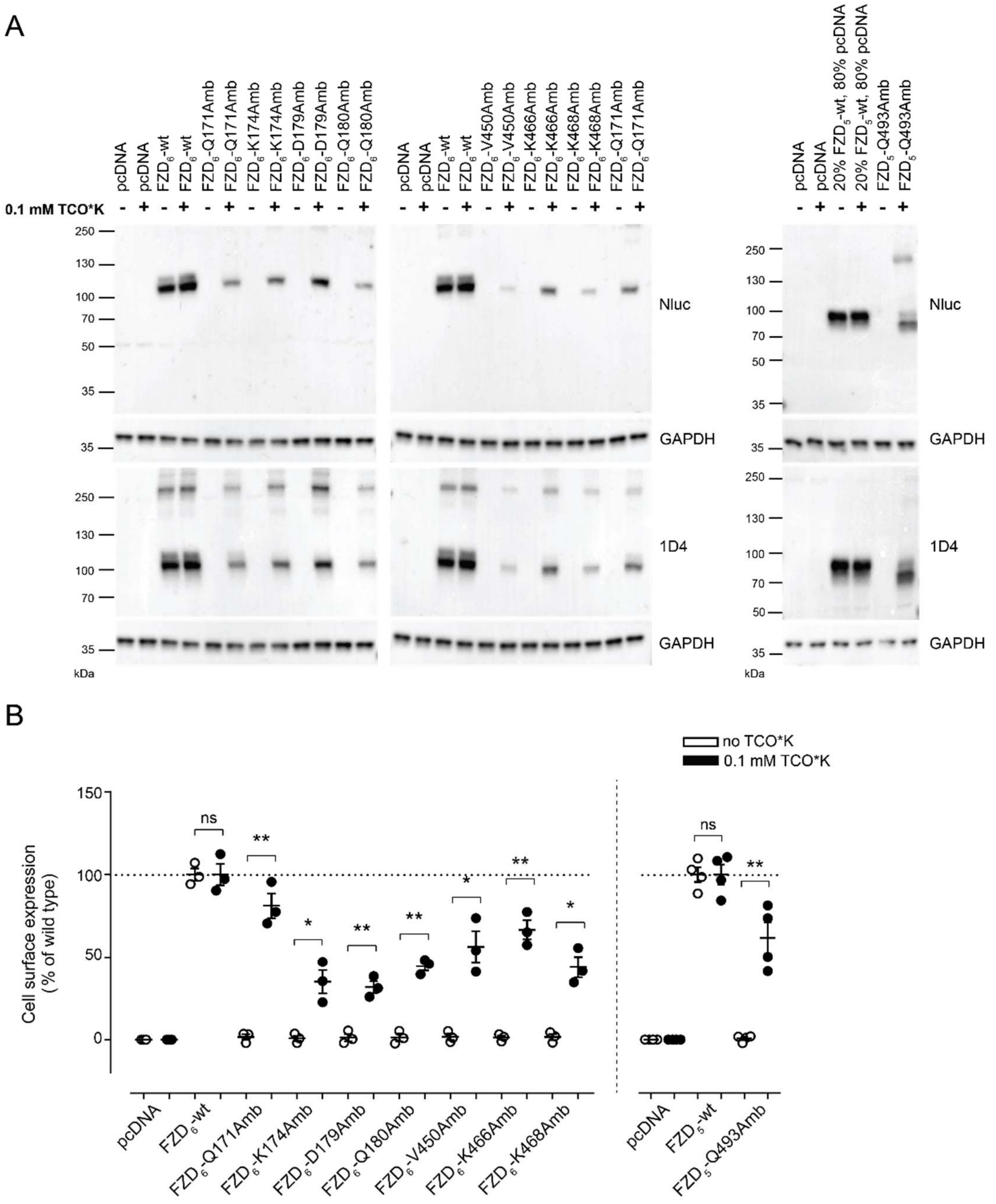
A
0.1 mM TCO*K
pcDNA
FZD6-wt
FZD6-Q171Amb
FZD6-K174Amb
FZD6-D179Amb
FZD6-Q180Amb
FZD6-V450Amb
FZD6-K466Amb
FZD6-K468Amb
20% FZD5-wt, 80% pcDNA
FZD5-Q493Amb
Nluc
GAPDH
1D4
kDa
B
no TCO*K
0.1 mM TCO*K
Cell surface expression (% of wild type)
ns
FZD5-wt

Fig. S2. Characterization of FZD_5 and FZD_6 amber mutants.
(**A**) Amber suppression in HEK293T cells cotransfected with pcDNA3.1 (control), Nluc-FZD_5-wt, FZD_6-wt or the indicated amber mutants and the orthogonal tRNA/synthetase pair in absence (-) or presence (+) of 0.1 mM TCO*K. Cells were lysed and analyzed by immunoblotting using anti-1D4 (detection of the full-length receptor) and anti-Nluc (detection of the N-terminal Nluc tag) antibodies. Anti-GAPDH served as a loading control. Note that the amount of FZD_5-wt was reduced to 20 % of the amount of transfected FZD_5-Q493Amb mutant (FZD_5-wt was balanced with pcDNA). (**B**) Cell surface expression in HEK293T cells transiently cotransfected with pcDNA3.1 or the indicated FZD_5 and FZD_6 constructs and the orthogonal tRNA/synthetase pair in absence (-) or presence (+) of 0.1 mM TCO*K was quantified by whole-cell ELISA using an antibody against the N-terminal Nluc tag. Data show mean ± SEM of three to four independent experiments performed in triplicates. Background fluorescence detected in pcDNA-transfected HEK293T cells was subtracted from all data, and mean values were normalized to wt FZD_5 or FZD_6 surface expression. Results were analyzed with one-way ANOVA and uncorrected Fisher's LSD post-hoc test. Significance levels are given as * ($P < 0.05$), ** ($P < 0.01$), and *ns* (not significant). Amb, amber mutant; Nluc, NanoLuciferase; TCO*K, TCO-Lysine; wt, wild type.

Fig. S3

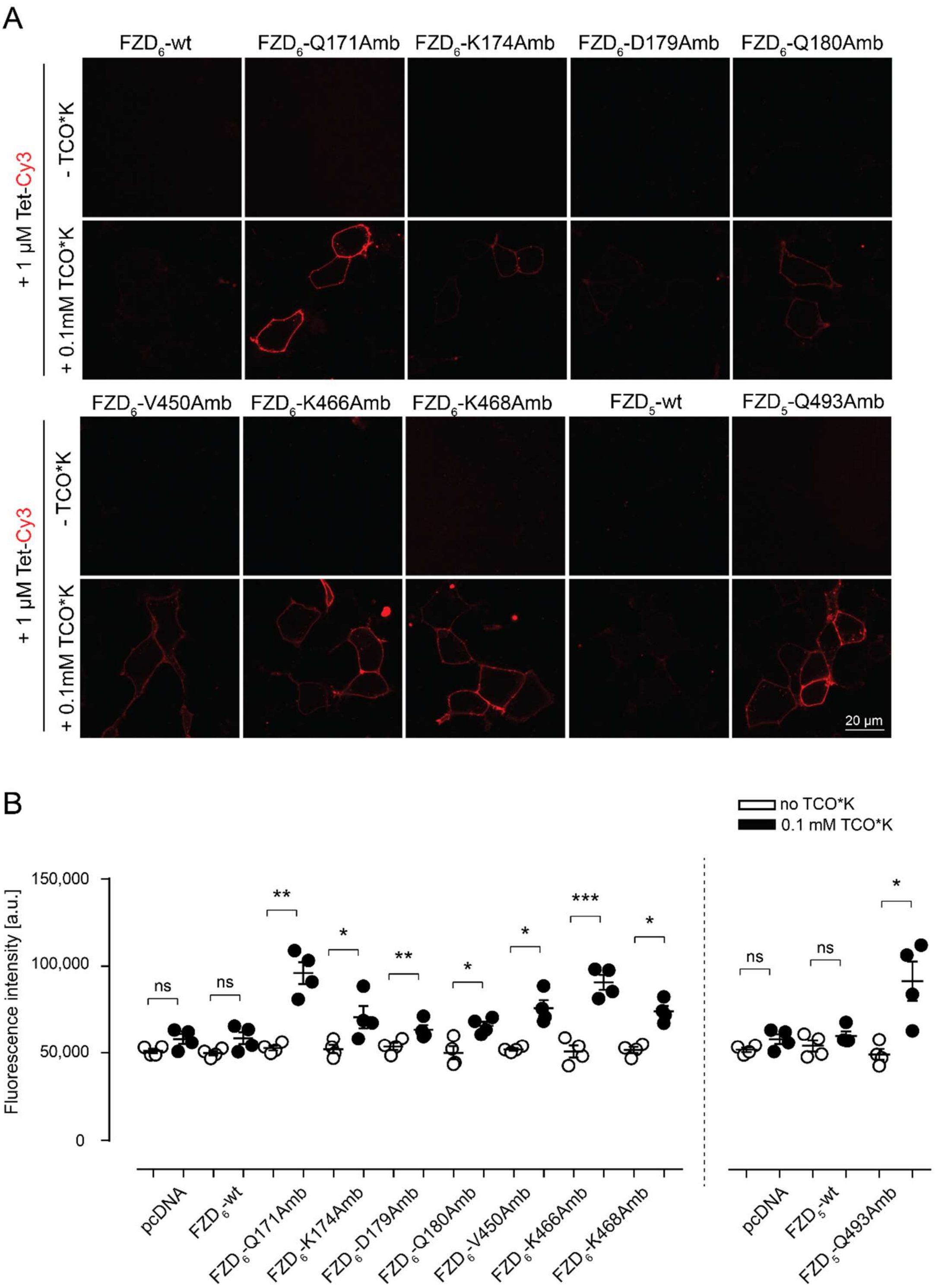
A
FZD6-wt
FZD6-Q171Amb
FZD6-K174Amb
FZD6-D179Amb
FZD6-Q180Amb
+ 1 µM Tet-Cy3
- TCO*K
+ 0.1mM TCO*K
FZD6-V450Amb
FZD6-K466Amb
FZD6-K468Amb
FZD5-wt
FZD5-Q493Amb
20 µm
B
no TCO*K
0.1 mM TCO*K
Fluorescence intensity [a.u.]
150,000
100,000
50,000
0
ns
**
*
***
pcDNA
FZD6-wt
FZD6-Q171Amb
FZD6-K174Amb
FZD6-D179Amb
FZD6-Q180Amb
FZD6-V450Amb
FZD6-K466Amb
FZD6-K468Amb
FZD5-wt
FZD5-Q493Amb

Fig. S3. Fluorescence labeling of FZD_5- and FZD_6-TCO*K-incorporated mutants with Tet-Cy3.

(**A**) Representative confocal images of HEK293T cells transiently cotransfected with FZD_5-wt, FZD_6-wt or the indicated amber mutants and the orthogonal tRNA/synthetase pair in absence (-) or presence (+) of 0.1 mM TCO*K. TCO*K-incorporated mutants were labeled with 1 µM of the cell membrane-impermeable fluorescent dye Tet-Cy3. Scale bar, 20 µm. (**B**) Fluorescence intensities of HEK293T cells transiently cotransfected with FZD_5-wt, FZD_6-wt or the indicated amber mutants and the orthogonal tRNA/synthetase pair in absence (-) or presence (+) of 0.1 mM TCO*K after labeling with 1 µM Tet-Cy3 measured in a plate reader assay. Data show mean ± SEM of four independent experiments performed in triplicates. Results were analyzed with one-way ANOVA and uncorrected Fisher's LSD post-hoc test. Significance levels are given as * ($P < 0.05$), ** ($P < 0.01$), *** ($P < 0.001$), and *ns* (not significant). Amb, amber mutant; Nluc, NanoLuciferase; TCO*K, TCO-Lysine; Tet, Tetrazine; wt, wild type.

Fig. S4

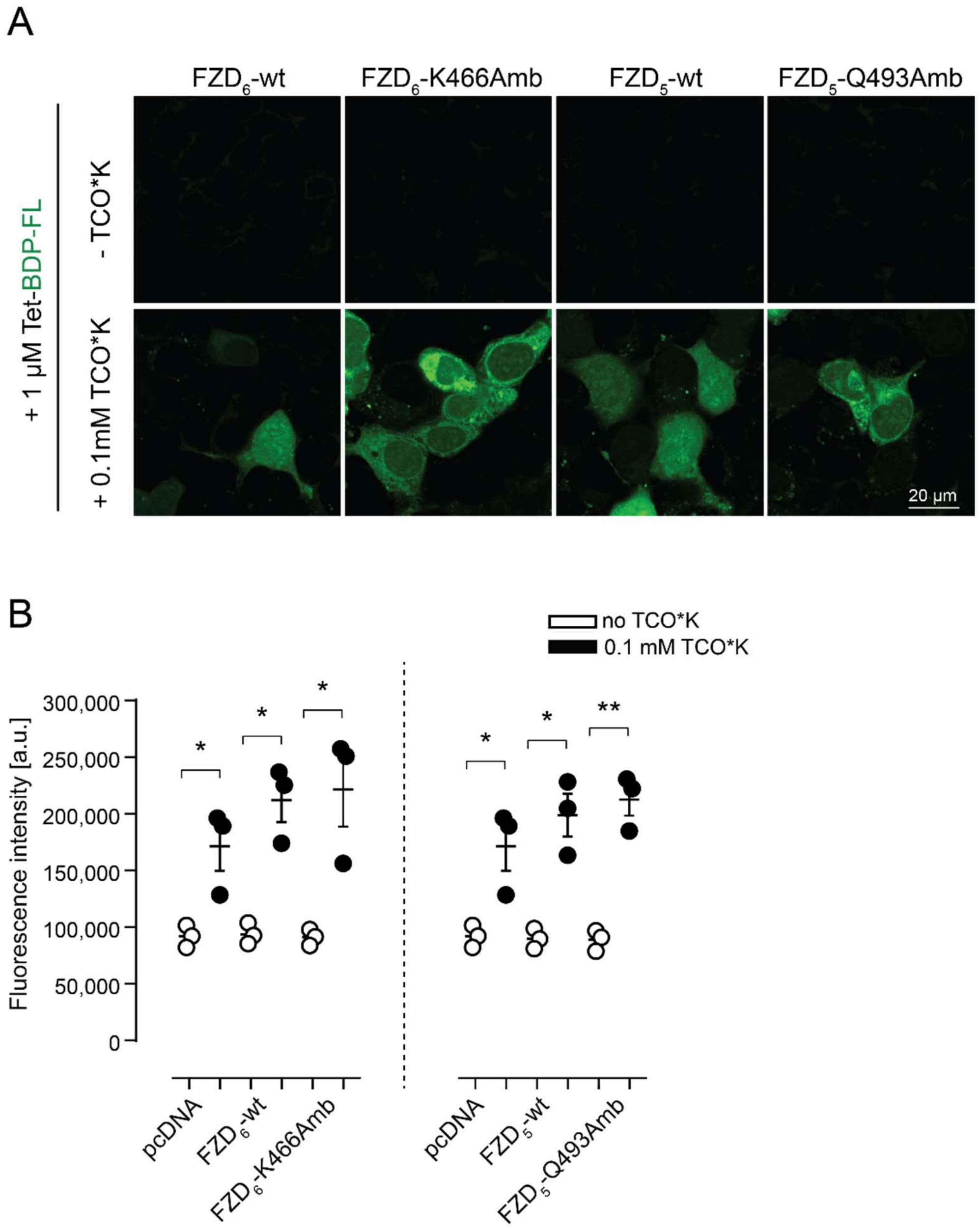
A
FZD6-wt
FZD6-K466Amb
FZD5-wt
FZD5-Q493Amb
+ 1 µM Tet-BDP-FL
- TCO*K
+ 0.1mM TCO*K
20 µm
B
no TCO*K
0.1 mM TCO*K
Fluorescence intensity [a.u.]
300,000
250,000
200,000
150,000
100,000
50,000
0
*
*
*
*
*
**
pcDNA
FZD6-wt
FZD6-K466Amb
pcDNA
FZD5-wt
FZD5-Q493Amb

Fig. S4. Fluorescence labeling of FZD_5- and FZD_6-TCO*K-incorporated mutants with Tet-BDP-FL.

(**A**) Representative confocal images of HEK293T cells transiently cotransfected with FZD_5-wt, FZD_6-wt or the indicated amber mutants and the orthogonal tRNA/synthetase pair in absence (-) or presence (+) of 0.1 mM TCO*K. TCO*K-incorporated mutants were labeled with 1 μM of the cell membrane-permeable fluorescent dye Tet-BDP-FL. Scale bar, 20 μm. (**B**) Fluorescence intensities of HEK293T cells transiently cotransfected with FZD_5-wt, FZD_6-wt or the indicated amber mutants and the orthogonal tRNA/synthetase pair in absence (-) or presence (+) of 0.1 mM TCO*K after labeling with Tet-BDP-FL measured in a plate reader assay. Data show mean ± SEM of three independent experiments performed in triplicates. Results were analyzed with one-way ANOVA and uncorrected Fisher's LSD post-hoc test. Significance levels are given as * ($P < 0.05$), and ** ($P < 0.01$). Amb, amber mutant; BDP-FL, BODIPY-fluorescein; Nluc, NanoLuciferase; TCO*K, TCO-Lysine; Tet, Tetrazine; wt, wild type.

Fig. S5

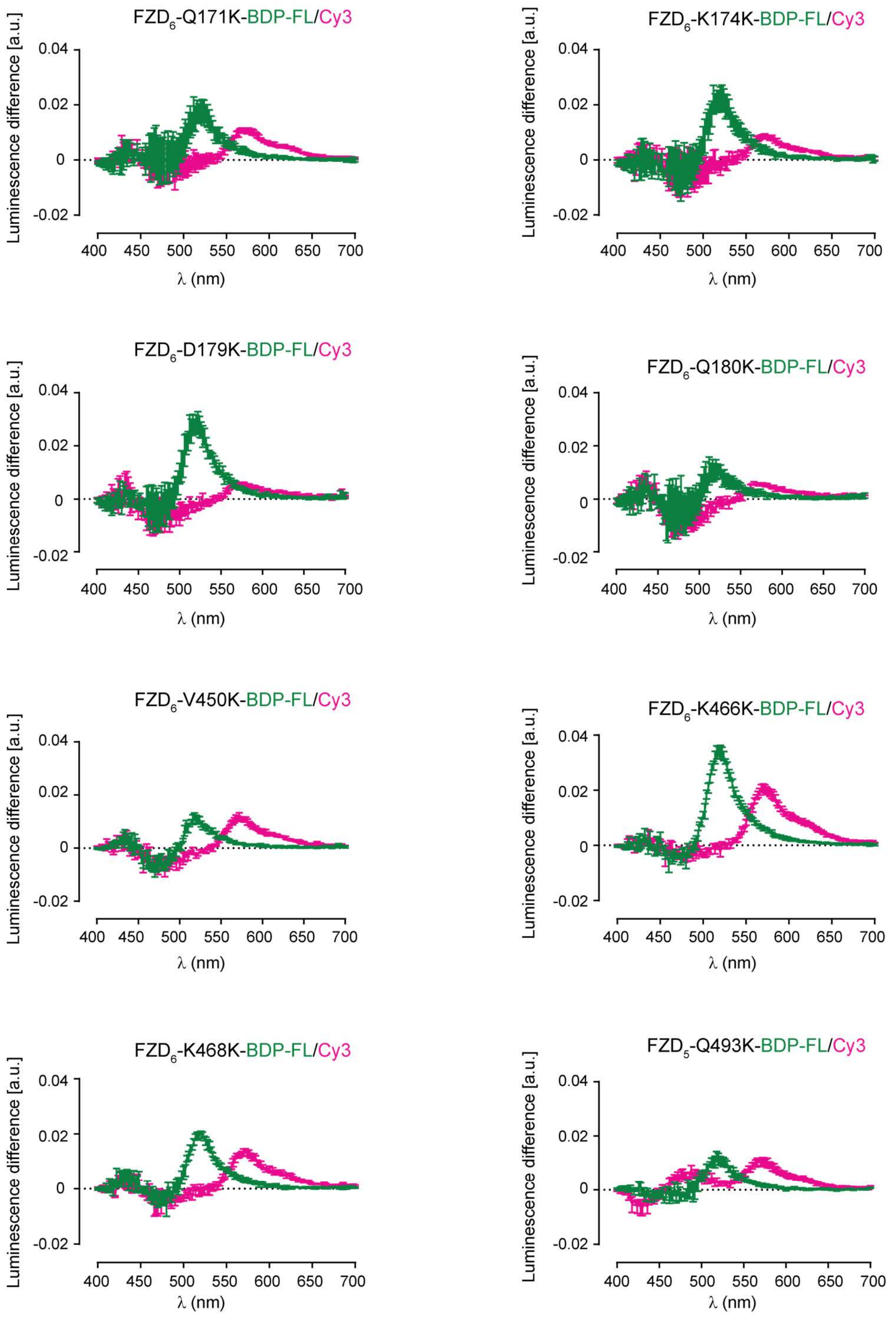

FZD$_6$-Q171K-BDP-FL/Cy3
FZD$_6$-K174K-BDP-FL/Cy3
FZD$_6$-D179K-BDP-FL/Cy3
FZD$_6$-Q180K-BDP-FL/Cy3
FZD$_6$-V450K-BDP-FL/Cy3
FZD$_6$-K466K-BDP-FL/Cy3
FZD$_6$-K468K-BDP-FL/Cy3
FZD$_5$-Q493K-BDP-FL/Cy3
Luminescence difference [a.u.]
0.04
0.02
0
-0.02
400 450 500 550 600 650 700
λ (nm)

Fig. S5. Comparison of different fluorescent dyes linked to FZD_5 and FZD_6 functioning as extracellular conformational BRET acceptors.

Luminescence emission spectra of BDP-FL (green)- and Cy3 (magenta)-labeled Nluc-$FZD_{5/6}$-TCO*K mutants and $FZD_{5/6}$-wt (no fluorescence labeling) were measured from 400 – 700 nm. The obtained spectra were normalized to the maximum of each replica (i.e., the Nluc emission peak around 450 nm). Luminescence obtained in FZD_5-wt or FZD_6-wt sensors (no TCO*K incorporated, no fluorescence labeling) was subtracted from the emission spectra of each receptor mutant to correct for unspecific binding of the fluorescent dyes in three independent experiments. All experiments were performed in HEK293T cells cotransfected with the indicated FZD_5 or FZD_6 sensors and the orthogonal tRNA/synthetase pair in presence of 0.1 mM TCO*K. BDP-FL, BODIPY-fluorescein; Nluc, NanoLuciferase; wt, wild type.

Fig. S6

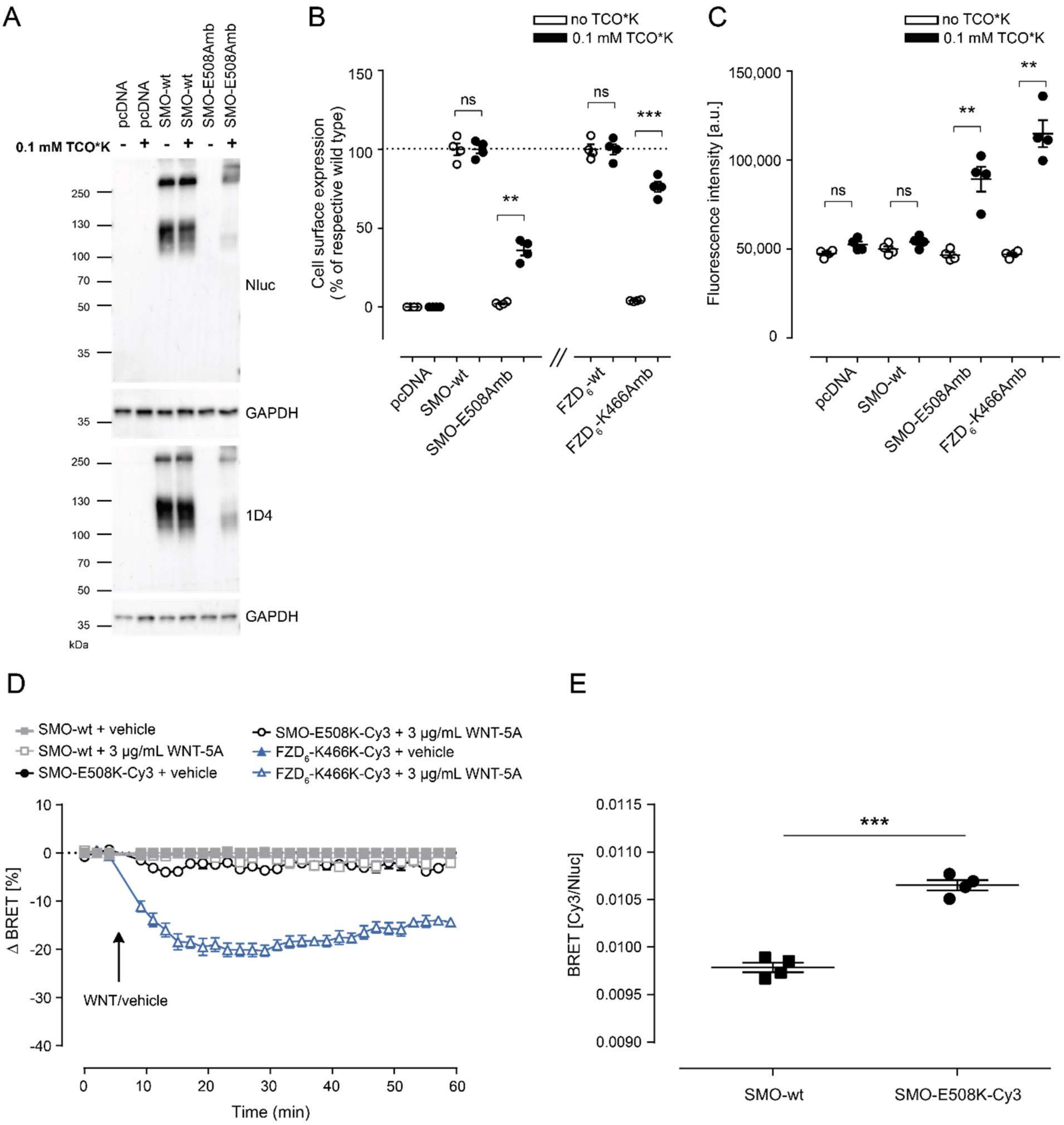
A
pcDNA
pcDNA
SMO-wt
SMO-wt
SMO-E508Amb
SMO-E508Amb
0.1 mM TCO*K
Nluc
GAPDH
1D4
GAPDH
kDa
B
no TCO*K
0.1 mM TCO*K
Cell surface expression (% of respective wild type)
ns
**
ns
***
pcDNA
SMO-wt
SMO-E508Amb
FZD6-wt
FZD6-K466Amb
C
Fluorescence intensity [a.u.]
150,000
100,000
50,000
SMO-E508Amb
FZD6-K466Amb
D
SMO-wt + vehicle
SMO-wt + 3 µg/mL WNT-5A
SMO-E508K-Cy3 + vehicle
SMO-E508K-Cy3 + 3 µg/mL WNT-5A
FZD6-K466K-Cy3 + vehicle
FZD6-K466K-Cy3 + 3 µg/mL WNT-5A
Δ BRET [%]
WNT/vehicle
Time (min)
E
BRET [Cy3/Nluc]
SMO-wt
SMO-E508K-Cy3

Fig. S6. A SMO CRD sensor confirms the specificity of the WNT-induced BRET signal. (**A**) Amber suppression in HEK293T cells cotransfected with pcDNA3.1 (control), Nluc-SMO-wt or the Nluc-mSMO-E508 amber mutant and the orthogonal tRNA/synthetase pair in absence (-) or presence (+) of 0.1 mM TCO*K. Cells were lysed and analyzed by immunoblotting using anti-1D4 (detection of the full-length receptor) and anti-Nluc (detection of the N-terminal Nluc tag) antibodies. Anti-GAPDH served as a loading control. (**B**) Cell surface expression in HEK293T cells transiently cotransfected with pcDNA3.1 or the indicated SMO constructs and the orthogonal tRNA/synthetase pair in absence (-) or presence (+) of 0.1 mM TCO*K was quantified by whole-cell ELISA using an antibody against the N-terminal Nluc tag. Data show mean ± SEM of four independent experiments performed in triplicates. Background fluorescence detected in pcDNA-transfected HEK293T cells was subtracted from all data, and mean values were normalized to wt SMO or wt FZD_6 surface expression. (**C**) Fluorescence intensities in HEK293T cells transiently cotransfected with pcDNA3.1, SMO-wt, SMO-E508Amb or FZD_6-K466Amb and the orthogonal tRNA/synthetase pair in absence (-) or presence (+) of 0.1 mM TCO*K after labeling with 1 μM Tet-Cy3 measured in a plate reader assay. Data show mean ± SEM of four independent experiments performed in triplicates. All results in (**B**) and (**C**) were analyzed with one-way ANOVA and uncorrected Fisher's LSD post-hoc test. Significance levels are given as ** ($P < 0.01$), *** ($P < 0.001$), and ns (not significant). (**D**) BRET responses of SMO and FZD_6-K466K-Cy3 CRD sensors upon 3 μg/mL WNT-5A treatment or vehicle control. The arrow indicates the time point of WNT/vehicle application. (**E**) Basal BRET ratio of Nluc-SMO-wt in comparison to Nluc-SMO-E508K-Cy3 of data shown in (**D**). Differences of the mean values ± SEM of the first three measurements before WNT/vehicle application were analyzed with Student's unpaired t-test. Significance level is given as *** ($P < 0.001$). Amb, amber mutant; CRD, cysteine-rich domain; Nluc, NanoLuciferase; TCO*K, TCO-Lysine; wt, wild type.

Fig. S7

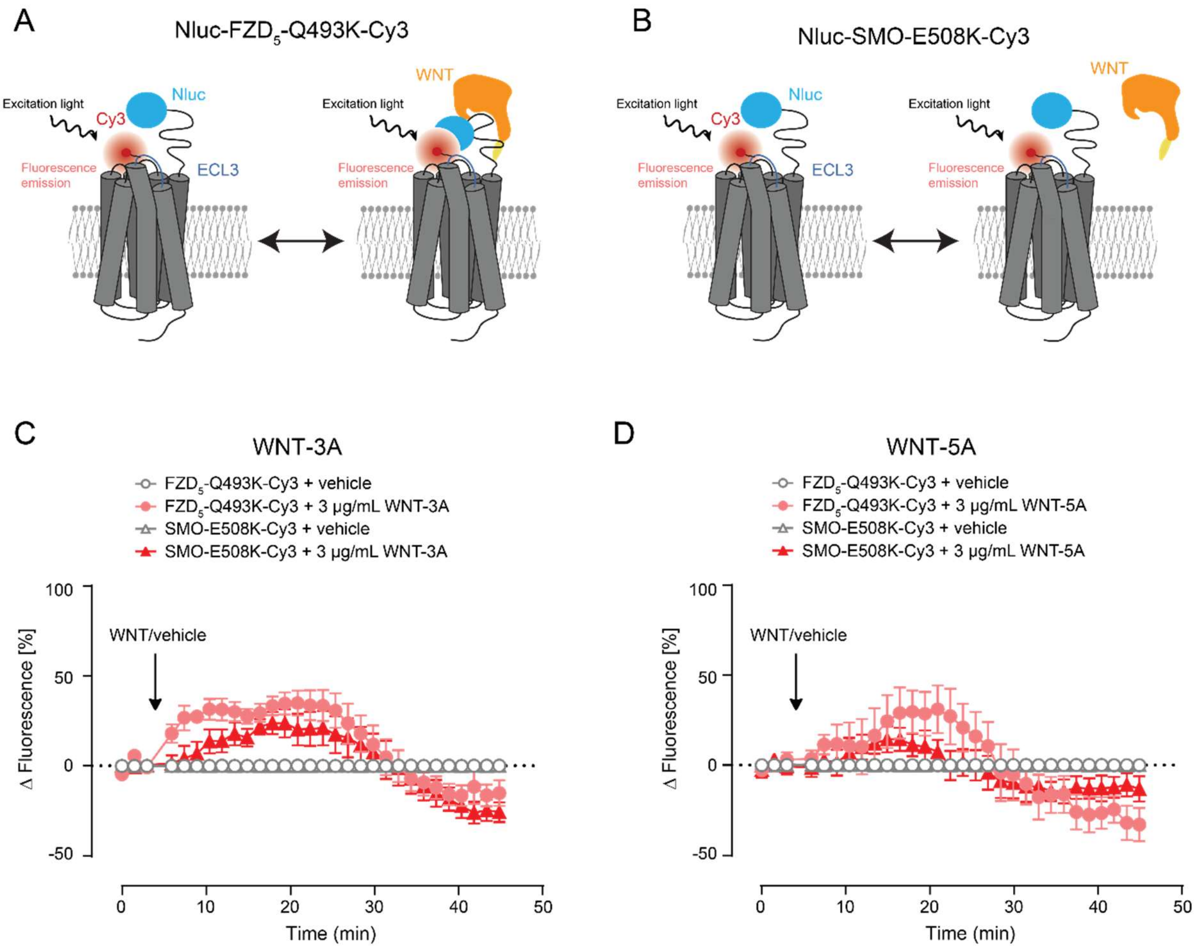
A
Nluc-FZD5-Q493K-Cy3
Excitation light
Cy3
Nluc
Fluorescence emission
ECL3
WNT
B
Nluc-SMO-E508K-Cy3
C
WNT-3A
FZD5-Q493K-Cy3 + vehicle
FZD5-Q493K-Cy3 + 3 μg/mL WNT-3A
SMO-E508K-Cy3 + vehicle
SMO-E508K-Cy3 + 3 μg/mL WNT-3A
WNT/vehicle
Δ Fluorescence [%]
Time (min)
D
WNT-5A
FZD5-Q493K-Cy3 + vehicle
FZD5-Q493K-Cy3 + 3 μg/mL WNT-5A
SMO-E508K-Cy3 + vehicle
SMO-E508K-Cy3 + 3 μg/mL WNT-5A

Fig. S7. ΔBRET is independent from a change in the environment of Cy3 fluorophores. (**A-B**) Schematic depiction of the extracellular sensors FZD_5-Q493K-Cy3 (**A**) and SMO-E508K-Cy3 (**B**) for fluorescence measurements upon WNT stimulation in the absence of an Nluc substrate. In contrast to FZD_5, SMO is not binding WNTs. (**C-D**) Fluorescence intensities of FZD_5-Q493K-Cy3 and SMO-E508K-Cy3 CRD sensors upon 3 μg/mL WNT-3A (**C**) and WNT-5A (**D**) treatment or vehicle control of six independent experiments. The arrow indicates the time point of WNT/vehicle application. All experiments were performed in HEK293T cells cotransfected with the indicated FZD_5 or SMO sensor and the orthogonal tRNA/synthetase pair in presence of 0.1 mM TCO*K and after labeling with 1 μM Tet-Cy3. ECL3, extracellular loop 3; Nluc, NanoLuciferase; TCO*K, TCO-Lysine; Tet, Tetrazine.

Fig. S8

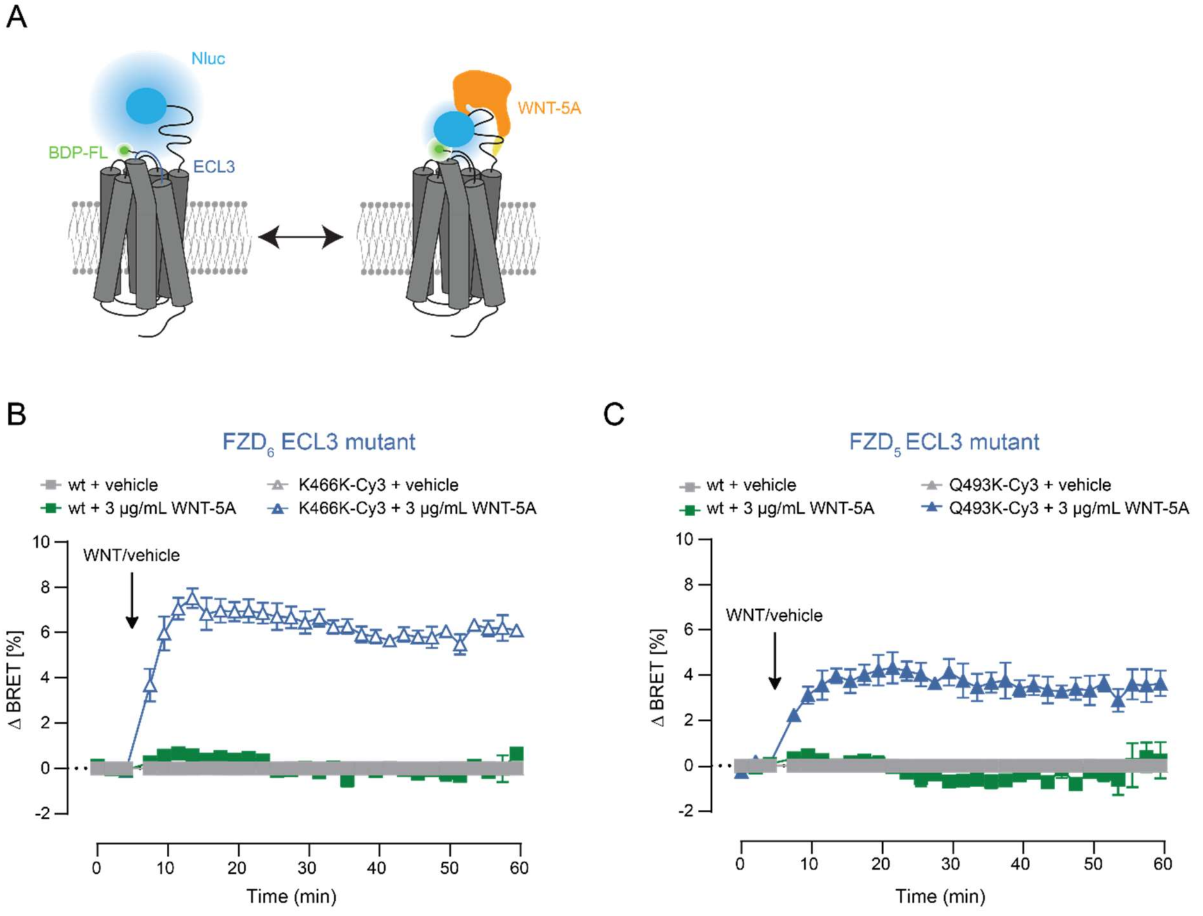
A
Nluc
WNT-5A
BDP-FL
ECL3
B
FZD6 ECL3 mutant
wt + vehicle
K466K-Cy3 + vehicle
wt + 3 µg/mL WNT-5A
K466K-Cy3 + 3 µg/mL WNT-5A
WNT/vehicle
Δ BRET [%]
Time (min)
C
FZD5 ECL3 mutant
wt + vehicle
Q493K-Cy3 + vehicle
wt + 3 µg/mL WNT-5A
Q493K-Cy3 + 3 µg/mL WNT-5A
WNT/vehicle
Δ BRET [%]
Time (min)

Fig. S8. CRD rearrangements in BDP-FL-labeled FZD$_5$ and FZD$_6$ in response to WNT-5A.

(**A**) Schematic depiction of the CRD sensor design with the N-terminally Nluc-tagged FZD. Fluorescence labeling of residues in the ECL3 (blue) region of the receptor with 1 μM Tet-BDP-FL. (**B-C**) BRET responses of FZD$_6$ (**B**) or FZD$_5$ (**C**) ECL3 mutant CRD sensors upon 3 μg/mL WNT-5A treatment or vehicle control. The arrow indicates the time point of WNT/vehicle application. All experiments were performed in HEK293T cells cotransfected with the indicated FZD$_5$ or FZD$_6$ sensors and the orthogonal tRNA/synthetase pair in presence of 0.1 mM TCO*K and after labeling with 1 μM Tet-BPD-FL. BDP-FL, BODIPY-fluorescein; CRD, cysteine-rich domain; ECL3, extracellular loop 3; Nluc, NanoLuciferase; TCO*K, TCO-Lysine; Tet, Tetrazine; wt, wild type.

Fig. S9

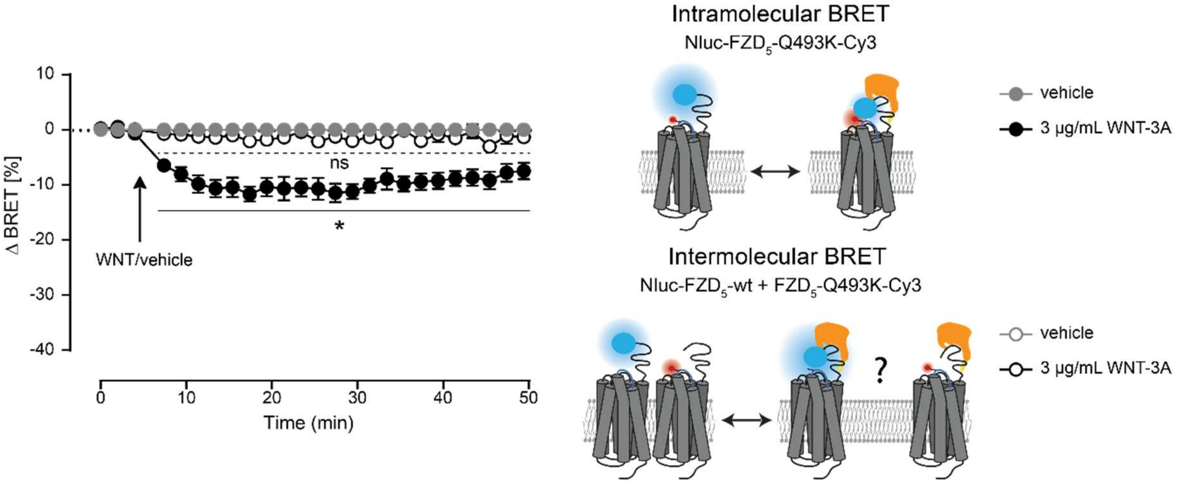

10
0
-10
-20
-30
-40
Δ BRET [%]
ns
*
WNT/vehicle
0
10
20
30
40
50
Time (min)
Intramolecular BRET
Nluc-FZD5-Q493K-Cy3
vehicle
3 µg/mL WNT-3A
Intermolecular BRET
Nluc-FZD5-wt + FZD5-Q493K-Cy3
?
vehicle
3 µg/mL WNT-3A

Fig. S9. Intra- *versus* intermolecular BRET responses in FZD_5.
BRET responses of HEK293T cells expressing Nluc-FZD_5-Q493K-Cy3 (intramolecular BRET) in comparison to an intermolecular BRET control where Nluc-FZD_5-wt is cotransfected with a Nluc-lacking FZD_5-Q493 amber mutant upon 3 µg/mL WNT-5A treatment or vehicle control of four independent experiments. All experiments were performed in HEK293T cells cotransfected with the indicated FZD_5 sensors and the orthogonal tRNA/synthetase pair in presence of 0.1 mM TCO*K and after labeling with 1 µM Tet-Cy3. The arrow indicates the time point of WNT/vehicle application. Differences between vehicle control and WNT-5A-induced BRET responses were analyzed with multiple t-test followed by Holm-Sidak multiple comparison. Significance levels are given as * (P < 0.05), and *ns* (not significant). Nluc, NanoLuciferase; TCO*K, TCO-Lysine; Tet, Tetrazine; wt, wild type.

Fig. S10

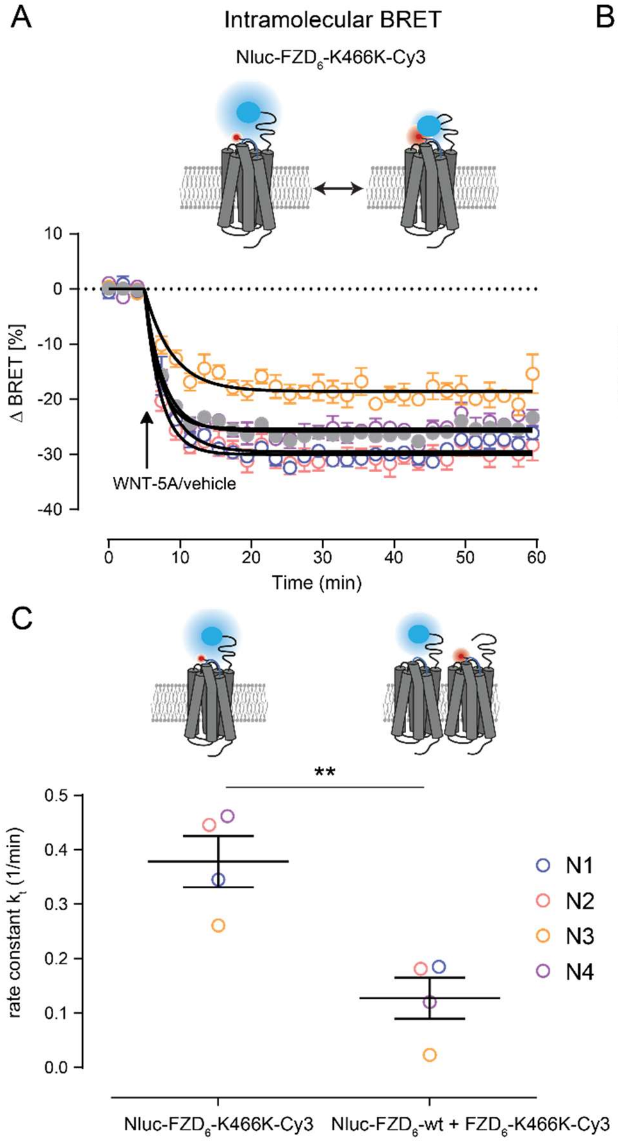
A
Intramolecular BRET
Nluc-FZD6-K466K-Cy3
Δ BRET [%]
WNT-5A/vehicle
Time (min)
B
Intermolecular BRET
Nluc-FZD6-wt + FZD6-K466K-Cy3
Δ BRET [%]
WNT-5A/vehicle
N1
N2
N3
N4
mean
Time (min)
C
**
rate constant k1 (1/min)
N1
N2
N3
N4
Nluc-FZD6-K466K-Cy3
Nluc-FZD6-wt + FZD6-K466K-Cy3

Fig. S10. Intra- *versus* intermolecular BRET responses in FZD$_6$.

(**A**) BRET responses of HEK293T cells expressing Nluc-FZD$_6$-K466K-Cy3 (intramolecular BRET) upon 3 µg/mL WNT-5A stimulation in four individual experiments (N1 – N4). (**B**) BRET responses of HEK293T cells expressing an intermolecular BRET control where Nluc-FZD$_6$-wt was cotransfected with a Nluc-lacking FZD$_6$-K466 amber mutant upon 3 µg/mL WNT-5A treatment or vehicle control. All experiments were performed in HEK293T cells cotransfected with the indicated FZD$_5$ or FZD$_6$ sensors and the orthogonal tRNA/synthetase pair in presence of 0.1 mM TCO*K and after labeling with 1 µM Tet-Cy3. The arrow indicates the time point of WNT application. (**C**) Rate constant k of Nluc-FZD$_6$-K466K-Cy3 (intramolecular BRET) and Nluc-FZD$_6$-wt + Nluc-lacking FZD$_6$-K466K-Cy3 intermolecular BRET control determined from fitted data in (**A**) and (**B**) by using the plateau followed by one phase decay equation. Differences of rate constant k of the Nluc-FZD$_6$-K466K-Cy3 CRD sensor and the Nluc-FZD$_6$-wt + FZD$_6$-K466K-Cy3 intermolecular BRET control were analyzed with Student's unpaired t-test. Significance level is given as ** ($P < 0.01$). Values in (**A**) to (**C**) represent mean ± SD of individual experiments (N1 – N4), mean ± SEM values of all four experiments are plotted in grey. CRD, cysteine-rich domain; Nluc, NanoLuciferase; TCO*K, TCO-Lysine; wt, wild type.

Fig. S11

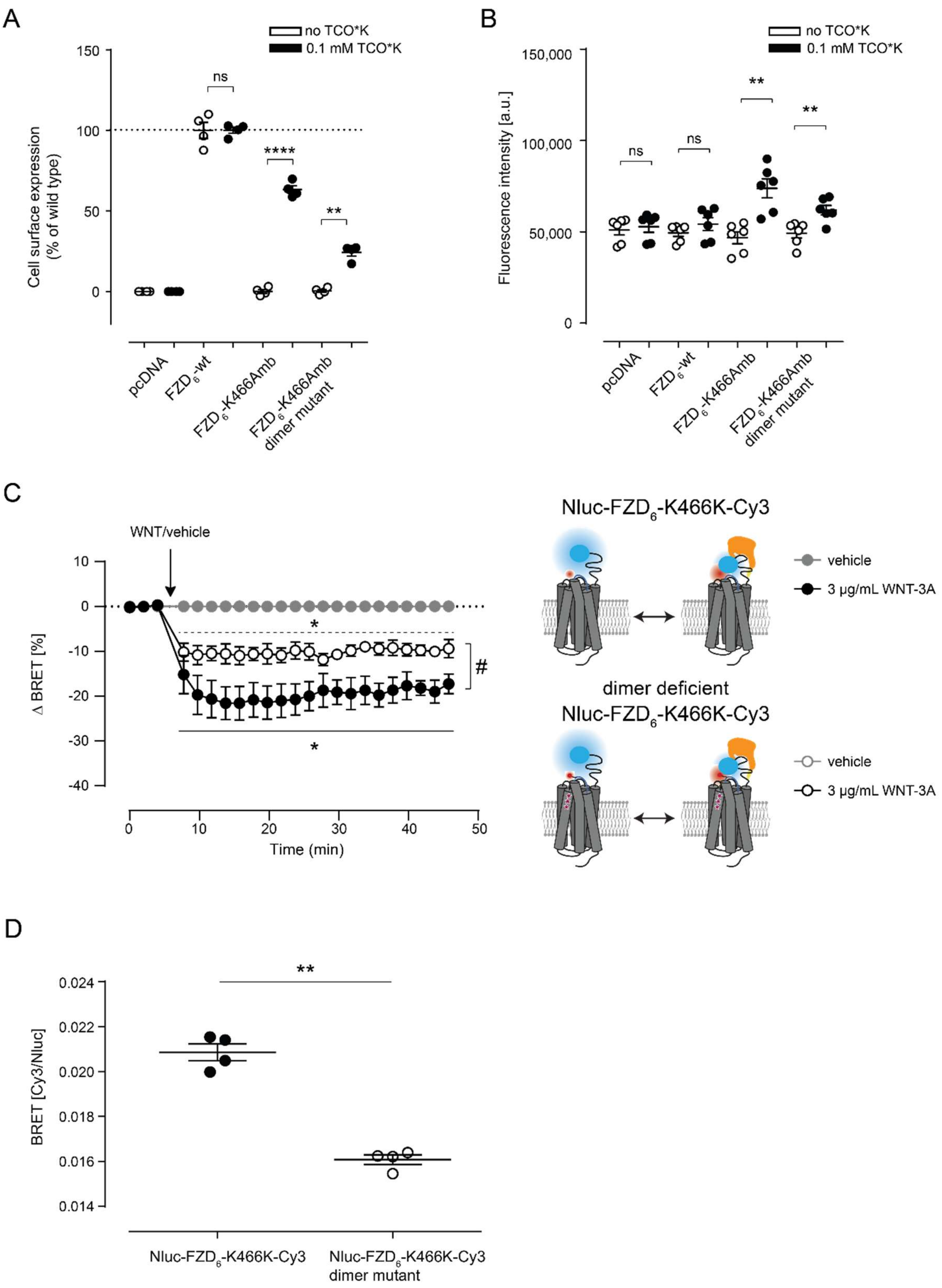
A
no TCO*K
0.1 mM TCO*K
Cell surface expression (% of wild type)
ns
****
**
pcDNA
FZD6-wt
FZD6-K466Amb
FZD6-K466Amb dimer mutant
B
Fluorescence intensity [a.u.]
150,000
100,000
50,000
C
WNT/vehicle
Δ BRET [%]
Time (min)
#
Nluc-FZD6-K466K-Cy3
dimer deficient Nluc-FZD6-K466K-Cy3
vehicle
3 µg/mL WNT-3A
D
BRET [Cy3/Nluc]
Nluc-FZD6-K466K-Cy3
Nluc-FZD6-K466K-Cy3 dimer mutant

Fig. S11. FZD_6-K466Amb dimer mutant controls for monomeric WNT-induced BRET responses.
(**A**) Cell surface expression in HEK293T cells transiently cotransfected with pcDNA3.1 or the indicated FZD_6 constructs with special focus on the FZD_6 dimerization deficient triple Ala mutant FZD_6-K466Amb-D365A/R368A/Y369A (named as FZD_6-K466Amb/K-Cy3 dimer mutant) and the orthogonal tRNA/synthetase pair in absence (-) or presence (+) of 0.1 mM TCO*K was quantified by whole-cell ELISA using an antibody against the N-terminal Nluc tag. Data show mean ± SEM of four independent experiments performed in triplicates. Background fluorescence detected in pcDNA-transfected HEK293T cells was subtracted from all data, and mean values were normalized to wt FZD_6 surface expression. (**B**) Fluorescence intensities of HEK293T cells transiently cotransfected with pcDNA3.1 or the indicated FZD_6 constructs and the orthogonal tRNA/synthetase pair in absence (-) or presence (+) of 0.1 mM TCO*K after labeling with Tet-Cy3 measured in a plate reader assay. Data show mean ± SEM of six independent experiments performed in triplicates. All results in (**A**) and (**B**) were analyzed with one-way ANOVA and uncorrected Fisher's LSD post-hoc test. Significance levels are given as ** ($P < 0.01$), **** ($P < 0.0001$), and *ns* (not significant). (**C**) BRET responses of HEK293T cells expressing the Nluc-FZD_6-K466K-Cy3 or the Nluc-FZD_6-K466K-Cy3 dimer mutant upon 3 µg/mL WNT-5A stimulation in four independent experiments. Differences between vehicle control and WNT-5A-induced BRET responses were analyzed with multiple t-test followed by Holm-Sidak multiple comparison. Significance level is given as * ($P < 0.05$). Plateaus of WNT-5A induced BRET responses of the two different sensors were analyzed with extra sum of squares F test. Significance level is given as # ($P < 0.05$). The arrow indicates the time point of WNT/vehicle application. (**D**) Basal BRET ratio of data shown in (**C**). Differences of the mean values ± SEM of the first three measurements before WNT/vehicle application were analyzed with Student's unpaired t-test. Significance level is given as ** ($P < 0.01$). Amb, amber mutant; Nluc, NanoLuciferase; TCO*K, TCO-Lysine; wt, wild type.

Fig. S12

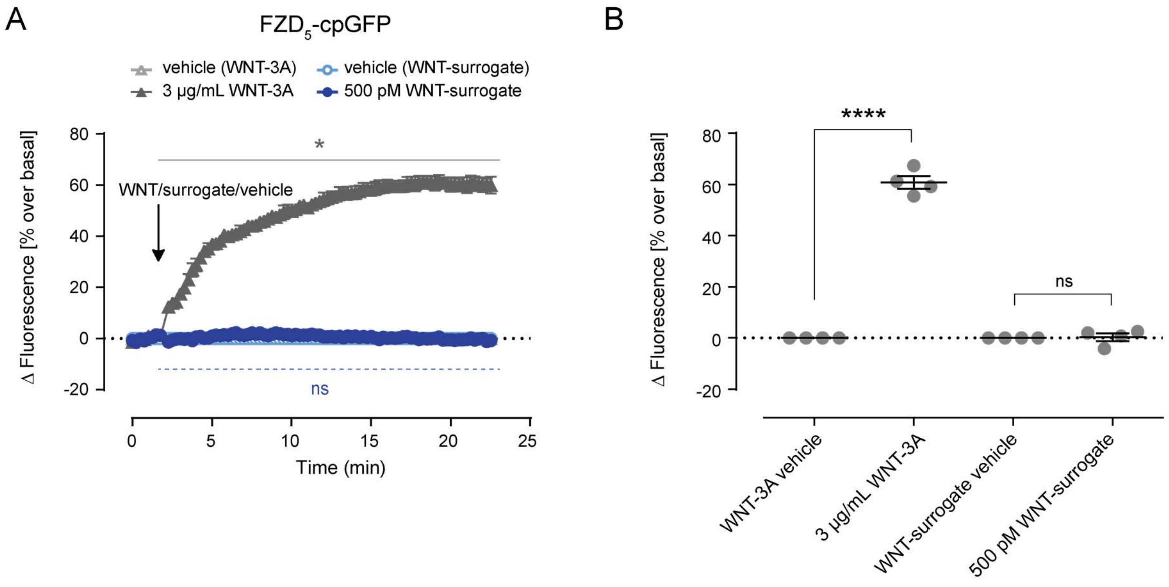
A
FZD5-cpGFP
vehicle (WNT-3A)
3 µg/mL WNT-3A
vehicle (WNT-surrogate)
500 pM WNT-surrogate
Δ Fluorescence [% over basal]
80
60
40
20
0
-20
*
WNT/surrogate/vehicle
ns
0
5
10
15
20
25
Time (min)
B
Δ Fluorescence [% over basal]
80
60
40
20
0
-20
****
ns
WNT-3A vehicle
3 µg/mL WNT-3A
WNT-surrogate vehicle
500 pM WNT-surrogate

Fig. S12. WNT-surrogate does not induce a response at the intracellular cpGFP biosensor.

(**A**) WNT-3A- and WNT-surrogate-induced fluorescence responses of HEK293A cells stably transfected with FZD_5-cpGFP of four independent experiments. The arrow indicates the time point of WNT/WNT-surrogate/vehicle application. Differences between vehicle control and WNT-3A- or vehicle control and WNT-surrogate-induced fluorescence responses were analyzed with multiple t-test followed by Holm-Sidak multiple comparison. Significance levels are given as * ($P < 0.05$), and *ns* (not significant). (**B**) Maximal fluorescence responses from data shown in (**A**). The results were analyzed with one-way ANOVA followed by Sidak's post-hoc test. Significance levels are given as **** ($P < 0.0001$), and *ns* (not significant). cpGFP, circularly permutated GFP.

Table S1. Fitted ΔBRET amplitudes of all FZD_5 and FZD_6 sensors.

	ΔBRET amplitudes in % (plateau followed by one phase decay equation ± SEM)		
	**3 μg/mL WNT-5A**		**3 μg/mL WNT-3A**
	Cy3 labeling	BDP-FL labeling	Cy3 labeling
**FZD_6**			
Wild type	-2.323 ± 1.022	0.104 ± 0.047	-1.657 ± 0.209
Q171K-Cy3	-11.61 ± 0.512		
K174K-Cy3	-17.91 ± 0.654		
D179K-Cy3	-13.24 ± 0.423		
Q180K-Cy3	-14.88 ± 0.455		
V450K-Cy3	-18.16 ± 0.408		
K466K-Cy3	-26.40 ± 0.744[#]	6.371 ± 0.107	-26.30 ± 0.377[#]
K468K-Cy3	-20.22 ± 0.816		
**FZD_5**			
Wild type	-0.843 ± 0.269	-1.103 ± 0.231	-0.865 ± 0.213
Q493K-Cy3	-15.60 ± 0.227[##]	3.70 ± 0.101	-16.01 ± 0.311[##]

[#], [##], no significant difference between effect of WNT-5A and WNT-3A on FZD_6-K466K-Cy3 and FZD_5-Q493K-Cy3 as analyzed with Student's unpaired t-test.
